# “Genome-wide identification of bZIP gene family in Pearl millet and transcriptional profiling under abiotic stress, phytohormonal treatments; and functional characterization of *PgbZIP9*”

**DOI:** 10.3389/fpls.2024.1352040

**Published:** 2024-02-26

**Authors:** Deepak Kumar Jha, Jeky Chanwala, Preeti Barla, Nrisingha Dey

**Affiliations:** ^1^ Division of Plant and Microbial Biotechnology, Institute of Life Sciences, Bhubaneswar, India; ^2^ Regional Centre for Biotechnology, Faridabad, India

**Keywords:** pearl millet, bZIP transcription factors, abiotic stress, plant genomics, ABI5 (ABA insensitive 5)

## Abstract

Abiotic stresses are major constraints in crop production, and are accountable for more than half of the total crop loss. Plants overcome these environmental stresses using coordinated activities of transcription factors and phytohormones. Pearl millet an important C4 cereal plant having high nutritional value and climate resilient features is grown in marginal lands of Africa and South-East Asia including India. Among several transcription factors, the basic leucine zipper (bZIP) is an important TF family associated with diverse biological functions in plants. In this study, we have identified 98 bZIP family members (PgbZIP) in pearl millet. Phylogenetic analysis divided these *PgbZIP* genes into twelve groups (A-I, S, U and X). Motif analysis has shown that all the PgbZIP proteins possess conserved bZIP domains and the exon-intron organization revealed conserved structural features among the identified genes. Cis-element analysis, RNA-seq data analysis, and real-time expression analysis of *PgbZIP* genes suggested the potential role of selected *PgbZIP* genes in growth/development and abiotic stress responses in pearl millet. Expression profiling of selected *PgbZIPs* under various phytohormones (ABA, SA and MeJA) treatment showed differential expression patterns of *PgbZIP* genes. Further, PgbZIP9, a homolog of AtABI5 was found to localize in the nucleus and modulate gene expression in pearl millet under stresses. Our present findings provide a better understanding of *bZIP* genes in pearl millet and lay a good foundation for the further functional characterization of multi-stress tolerant *PgbZIP* genes, which could become efficient tools for crop improvement.

## 1 Introduction

Plants often become targets of various adverse environmental conditions, including biotic and abiotic stresses. Major abiotic stresses include salinity, extreme temperatures, and drought, while biotic stresses are pathogenic infections, insects, and weed attacks. Abiotic stresses negatively affect plant development, growth and survivability, reducing plant productivity (Ahmad and Prasad, 2011; Zhao et al., 2017). Drought increases the osmotic pressure which alters leaf morphology and size (Liang et al., 2022). It also induces poor seed germination and cell damage due to accumulation of reactive oxygen species or cellular dehydration, ultimately resulting in the death of parts of plant or entire plant (Sornaraj et al., 2016; Liang et al., 2022). Similarly, salt stress disrupts ionic homeostasis, producing ion toxicity and oxidative stress (Liang et al., 2018). In addition, salinity decreases the rate of photosynthesis (Van Zelm et al., 2020). All these unfavorable environmental conditions cause severe crop loss and according to the FAO report (2021), there is a loss of 34% of agricultural production amounting to a loss of USD 37 billion due to drought alone. Alongside, due to salinity, around 1 billion hectares of the arid and semi-arid regions are deserted, while 20% of irrigated lands are affected by secondary salinization globally (Sahab et al., 2021) and it is expected that by 2050, up to 50% of arable land will be lost (Wang et al., 2003). Under these scenarios, environmentally stress-resilient crop plants like millet can become a choice for alternate food resources in days to come.

Pearl millet (*Pennisetum glaucum)* is a C4 plant that belongs to the Poaceae family and grows in arid or semi-arid regions of sub-Saharan Africa and India (Debieu et al., 2017). It is considered an environment-friendly crop as it can be cultivated in infertile soil and withstand high temperatures, and drought. The NADP-dependent malic enzyme pathway enables pearl millet to utilize water efficiently under high temperatures (Pardo and Vanburen, 2021). Pearl millet has high carbohydrate (70% starch) and protein content (8-16%) (Pujar et al., 2020; Punia et al., 2021). It has higher nutritional value compared to other cereals like rice, maize, and wheat, thus can serve as food for humans, while feed and fodder for animals (Dias-Martins et al., 2018). It also contains essential micronutrients such as iron and zinc (Krishnan and Meera, 2018). Pearl millet has natural adaptation against elevated drought and heat stress. We assume by 2050 world population is expected to reach 10 billion (Searchinger et al., 2019); however, due to climatic abnormalities major crops such as rice, wheat, and maize may fail to grow as they are not tolerant to environmental fluctuations. On this backdrop, we assume pearl millet has high potential to become a mainstream food crop in the recent future; for the same the year 2023 has been observed as the year of millets (Poshadri et al., 2023).

Plants have developed complex mechanisms to defend themselves against environmental stresses through signaling pathways in which transcription factors (TFs) are significant players among many others (Ben Rejeb et al., 2014; Viswanath et al., 2023). Transcription factors are multi-domain proteins that bind DNA and subsequently recruit other proteins to initiate transcription. Recent studies on various TF families have identified DREB/CBF, bZIP, HD-Zip, GRAS, WXPL TF, MYB, WRKY, SHN, NAC, and other transcription factors in plants (Erpen et al., 2018; Khan et al., 2018; Chanwala et al., 2020; Hrmova and Hussain, 2021; Jha et al., 2021; Chanwala et al., 2022). These important regulatory transcription factors are associated with stress responses through either ABA-dependent or ABA-independent signaling pathways (Wani et al., 2021; Strader et al., 2022). TFs enhance or suppress the expression of downstream effector genes by cross-talk with phytohormones such as salicylic acid (SA), gibberellic acid (GA), abscisic acid (ABA), jasmonic acid (JA), brassinosteroids (BR), cytokinin (CK), ethylene (C_2_H_4_), and strigolactone (SL) for stress-mediated responses (Waadt et al., 2022). In a recent study, Hu et al. showed that phytohormone like jasmonic acid upregulates the genes acting in the CBF/DREB1 signaling (Hu et al., 2013). In addition, the exogenous application of JA increases the cold tolerance of plants (Hu et al., 2013). Similarly, the ABA cascade is crucial against most abiotic stimuli, primarily against drought stress (Waadt et al., 2022). The bZIP-type TFs such as ABRE- binding factors/ABRE-binding proteins (ABFs/AREBs), bind to major cis-acting element, ABA- RESPONSIVE ELEMENT (ABRE) and regulate the genes responsible for drought response (Chen et al., 2020b).

Basic leucine zipper (bZIP) is one of the largest families of TFs in the plant. They are made up of α helix and have two characteristic structural features: a sixteen amino acid long basic region and a repeat of seven leucine residues (Jakoby et al., 2002). The basic region is relatively conserved and contains a nuclear localization signal as well as a DNA binding N-x_7_ -R/K motif (Ye et al., 2023). However, the leucine zipper region is comparatively variable and consists of several hydrophobic amino acids (Jakoby et al., 2002). The hydrophobic sides of leucine repeat and N-x_7_ -R/K motif interact with each other to form a zipper-like structure (Dröge-Laser et al., 2018). Like other transcription factor families, bZIP genes also have diverse roles in biological functions such as plant growth, fruit development, seed maturation, senescence, stress response and photoreception (Alves et al., 2013; Sornaraj et al., 2016; Zhang et al., 2021). The bZIP TFs play critical role in defense response against various abiotic stress such as salinity and drought stress (Wang et al., 2021), cold stress (Hwang et al., 2016), and heat (Huang et al., 2021). Till now, there are number of bZIP members identified in various plant species. For instance, 78 members in *Arabidopsis* (Dröge-Laser et al., 2018), 247 BnbZIP in rapeseed (Zhou et al., 2017), 65 StbZIP in potato (Zhao et al., 2020), 88 JrbZIP in walnut (Quan et al., 2019), 54 LcbZIPs in litchi (Hou et al., 2022), 62 bZIP gene in Chinese pear (Manzoor et al., 2021), 119 BolbZIP in cabbage (Hwang et al., 2016), 50 JcbZIP in *Jatropha curcas* (Wang et al., 2021). However, there are no reports on expression profiling of the bZIP TF family and elucidating their functional role in pearl millet under stresses.

In this nascent study, we performed genome-wide identification of bZIP TFs in pearl millet followed by *in-silico* characterization, including phylogeny analysis, chromosomal mapping, motif and gene structure organization. Parallelly, the transcriptome analysis and relative expression assay of candidate genes under different abiotic stresses (drought, heat and salt) and phytohormonal treatment (ABA, SA and MeJA) carried out. Moreover, bZIP transcription factors impart stress responses through ABA mediated signaling, where *ABA-insensitive* (*ABI*) genes namely *ABI3*, *ABI4* and *ABI5* play very crucial role (Brocard et al., 2002). *ABI5*, a bZIP transcription factor forms the core of ABA signaling and regulate the expression of downstream functional genes having ABA response element (ABRE) in their promoter region (Skubacz et al., 2016). Therefore our study further inclined toward functional analysis of ABI5 encoding *PgbZIP9*, a multi-stress-responsive candidate gene. Insight of this study will provide a better understanding of the molecular function of the bZIP TF family in response to different environmental stresses in crop plants.

## 2 Materials and method

### 2.1 Plant materials

For this study, PRLT 2/89-33, a pearl millet germplasm was received from the International Crops Research Institute for Semiarid Tropics (ICRISAT) Patancheru, India. The mixture of red and black soil (1:1) was used for the multiplication of pearl millet. They were grown in a light/dark cycle of 16/8h at 28°C ( ± 2).

### 2.2 Identification of bZIP TFs in pearl millet and sequence analysis

The whole pearl millet genome and proteome dataset, along with GFF (General feature format file) were obtained from the pearl millet genome database (http://cegsb.icrisat.org/ipmgsc/) (Varshney et al., 2017). The protein sequences of bZIP TFs of *Arabidopsis thaliana* (79), *Oryza sativa* L. (89), and *Seteria italic* (90) were extracted from TAIR (https://www.arabidopsis.org/tools/bulk/protein/index.jsp) (Lamesch et al., 2012), Oryzabase (Kurata and Yamazaki, 2006) and Phytozome database (https://phytozome.jgi.doe.gov/pz/portal.html) (Goodstein et al., 2012) respectively. The total of above bZIP sequences (258) was used as a reference for the identification of bZIP TF family in pearl millet. Initially, these bZIP sequences were aligned, and hidden Markov model (HMM) profile was built by employing hmmbuild program of the HMMER tool v3.2, then hmmsearch was performed against the pearl millet proteome dataset to identify putative bZIP proteins (Finn et al., 2011). HMM files of proteins having bZIP_1 (PF00170), bZIP_Maf (PF03131), bZIP_C (PF12498) and bZIP_2 (PF07716) were retrieved from Pfam (http://pfam.xfam.org/) (Finn et al., 2014) and inspection of the identified sequences was done against them. Further, the presence of these domains in the probable pearl millet bZIP sequence was confirmed through either NCBI CDD (http://www.ncbi.nlm.nih.gov/Structure/cdd/wrpsb.cgi) (Marchler-Bauer et al., 2015), SMART (http://smart.embl-heidelberg.de/) (Letunic et al., 2021), or HMMScan (https://www.ebi.ac.uk/Tools/hmmer/search/hmmscan) (Finn et al., 2011). All the non-redundant hits were taken and redundant sequences having cut-off values greater than 0.01 were removed. Analysis of all the physical and chemical features of the identified bZIP protein was done on Expasy server using ProtParam tool (Gasteiger et al., 2005). Also, the Wolf PSORT (http://wolfpsort.org) (Horton et al., 2007) was employed to predict their subcellular position. The probability of pearl millet bZIP members being a transmembrane protein was indicated by employing the TMHMM-2.0 online tool (https://services.healthtech.dtu.dk/service.php?TMHMM-2.0) (Krogh et al., 2001).

### 2.3 Chromosomal mapping

For mapping the genomic coordinates of bZIP TF genes were obtained and they were located on all the chromosomes of pearl millet accordingly with the MapInspect software (http://mapinspect.software.informer.com/).

### 2.4 Phylogeny, motif and gene structure prediction

The bZIP protein sequences of *Arabidopsis*, rice, foxtail millet and pearl millet were taken for phylogenetic analysis. All the 356 bZIP protein sequences were aligned using MUSCLE with default parameters (Edgar, 2004). Then, MEGAV10.0 software (Kumar et al., 2018) was employed to generate the phylogenetic tree by using the maximum likelihood method with 1000 bootstrap replication and other parameters (Jones-Taylor-Thronton (JTT) model; partial deletion of gaps and rates among sites- gamma distribution, G).

The MEME suite program (Bailey et al., 2009) was employed to predict the conserved motifs in identified PgbZIP protein sequences considering the following parameters (maximum number of motifs- 20; minimum width- 6 and maximum width- 50. Then the obtained results were visualized by TBtools (Chen et al., 2020a). To analyze the gene structure of identified PgbZIP, intron/exon organization of each *bZIP* gene was predicted by the GSDS web server (Hu et al., 2015).

### 2.5 Gene duplication and synteny analysis

The duplication events of *PgbZIP* genes were assessed by the Multiple Collinearity Scan toolkit (MCScanX) with the default parameters (Wang et al., 2012). The synteny relationship between the *PgbZIP* genes and the *bZIP* genes from *A. thaliana, O. sativa*, and *S. italica* was visualized by AccuSyn software (Siri et al., 2020). The synonymous (Ks) and nonsynonymous (Ka) substitution rates were determined through PAL2NAL server (http://www.bork.embl.de/pal2nal) (Suyama et al., 2006).

### 2.6 Gene ontology annotation and cis-element analysis

The gene ontology annotation was performed using Blast2GO-OmicsBox v3.1 with default parameters (https://www.biobam.com/omicsbox) (Götz et al., 2008). GO terms were identified using mapping and InterProScan analysis. Furthermore, identified GO terms were used to predict the biological processes, cellular components and molecular functions associated with each PgbZIPs.

For cis-element analysis, 2000 bp upstream region of all the identified *PgbZIP* genes were obtained from the pearl millet genome database. These sequences were then uploaded into the PlantCARE server to identify regulatory cis-elements in the upstream region of *PgbZIP* genes (Rombauts et al., 1999).

### 2.7 Protein-protein interaction network analysis

The identified PgbZIP proteins were used as target and their orthologous *Arabidopsis* protein were employed as a reference to predict functional protein-protein interaction networks through the STRING website (https://string-db.org/) (Szklarczyk et al., 2022).

### 2.8 Transcriptome analysis

To look into the expression profile of *PgbZIP* genes, the RNAseq data of salt and drought treatment in pearl millet were acquired from the Sequence Read Archive (SRA) database of NCBI (https://www.ncbi.nlm.nih.gov/sra) (Shinde et al., 2018; Shivhare et al., 2020). Bowtie 2.0 (Langmead and Salzberg, 2012) was employed for alignment and mapping of obtained transcriptome dataset, then these RNA-seq reads were quantified by RSEM software (Li and Dewey, 2011). Finally, the differentially expressed genes were identified with an edgeR package (10.18129/B9.bioc.edgeR) (Robinson et al., 2010) and visualized with the help of TBtools software.

### 2.9 Stress treatment condition

For the analysis of tissue-specific expression patterns, pearl millet samples of leaves, stem, and root tissues were used. To look into the role of the *PgbZIP* genes under stress conditions, different abiotic stresses (drought, heat, salinity) and hormonal treatment (SA, MeJA, and ABA) were imposed on the four-week-old pearl millet seedlings. For drought stress, we withheld the irrigation of pearl millet seedlings for 8 days and rewatered on 9^th^ day for recovery stage expression pattern (Jha et al., 2021; Chanwala et al., 2023b). In case of salt stress, seedlings were placed in Hoagland solution containing 250 mM NaCl, whereas for control, seedlings were grown on half-strength Hoagland solution. Leaf samples were harvested from both control and treated plants at time points of 0, 12 and 24 h. For heat stress, pearl millet seedlings were kept at 45°C for 12h and samples were collected at 0, 3, and 12. To assess the effect of phytohormones, the pearl millet seedlings were spyayed with 100 μM Abscisic acid (ABA), 100 μM methyl jasmonate (MeJA), and 100 μM salicylic acid (SA). For control water was sprayed. Samples were harvested at 0h, 3h (early) and 24h (late) post treatment. All the collected samples were snap-frozen in liquid nitrogen and stored at –80°C until further analysis. All samples were collected in biological triplicates.

### 2.10 RNA extraction and expression analysis

Total RNA was isolated by using Spectrum Plant Total RNA kit (Sigma-Aldrich). RNA quality was checked on a 1.2% agarose gel with 18% formaldehyde. The purity and yield were estimated by NanoDrop ND-2000 spectrophotometer (Thermo Scientific) and RNA samples with 260/280 nm ratio between 2.0 to 2.1 were used for further analysis. RNA purification was done by treating with DNAse I (Sigma-Aldrich) as per the manufacturer’s protocol.

cDNA synthesis was done by first-strand cDNA synthesis kit (Thermo Scientific). All the quantitative real time-PCR (qRT-PCR) of selected *bZIP* genes were done in the QuantStudio™ 5 Real-Time PCR System (Applied Biosystems) and primers for this study were designed by Prime quest tool of IDT (Owczarzy et al., 2008). For quantitative real-time PCR, a 20 µL reaction mixture was used which contained 10 µL of SYBR Premix buffer (GoTaq^®^ qPCR MasterMix 2X Promega), 2 µL (20 ng) of cDNA, 1.0 µL each of forward and reverse primer (5 mM) and 6 µL of nuclease-free water. The quantitative real-time PCR run profile was as follows: 95°C for 10 min, followed by 40 cycles of 95°C for 15s, 60°C for 1 min. The transcript levels for individual genes were calculated by the 2^-ΔΔCT^ (CT-comparative threshold cycle) method and expressed as fold change in comparison to controls (Livak and Schmittgen, 2001). *Actin* and *ef1α* (Reddy et al., 2015) genes were taken for data normalization.

### 2.11 *In-silico* analysis of PgbZIP9

Homologous protein sequences of PgbZIP across species were collected from different sources, and these retrieved sequences were employed for sequence alignment through the Praline server(http://ibivu.cs.vu.nl/programs/pralinewww/) (Simossis and Heringa, 2003) and construction of phylogeny with the help of MEGA7. Also, the conserved bZIP domain prediction and gene-structure analysis were done by SMART and GSDS servers respectively. The structure of PgbZIP9 was predicted with the help of Alpha-fold (Jumper et al., 2021; Varadi et al., 2022).

### 2.12 Isolation and cloning of *PgbZIP9*

The candidate gene, *PgbZIP9* was isolated from the cDNA of pearl millet leaf and root samples using gene-specific primers. Then, it was cloned in pGEM^®^-T Easy Vector Systems (Promega). Further, the full-length gene with stop codon was cloned in the modified pCAMBIA-2300 for expression analysis in plants and in pCAMBIA-2300GFP without stop codon for subcellular localization study. Also, full-length *PgbZIP9* was sub-cloned into the yeast expression vector pGBKT7 for analyzing its transactivation activity.

### 2.13 Subcellular localization and transactivation assay of PgbZIP9

The PgbZIP9-GFP construct and empty pCAMBIA-2300GFP (control) were bombarded into onion epidermal cells by particle bombardment with a Bio-Rad Helios gene gun following the manufacturer’s protocol (Xiao et al., 2021). The fluorescence signals were visualized under a laser confocal scanning microscope (Leica, STED microscope).

For transactivation assay in yeast, the pGBKT7 clone of *PgbZIP9*, along with negative control (empty pGBKT7 vector), and positive control (pGBKT7-53) were transformed into Y2H Gold yeast strain (Clontech, USA) through the LiCl method. The transformed yeast colonies were streaked onto SD/-Trp medium and SD/-Trp/-His/-Ade medium with or without X-α-gal and allowed to grow at 30°C for 3-5 days (Cai et al., 2017).

### 2.14 Transient pearl millet transformation and stress treatment

Transient pearl millet transformation and stress assays in seedlings were performed following the procedures mentioned by Ji Xiaoyu et al. with slight modifications (Ji et al., 2014). The 7-days-old seedlings of pearl millet were transformed with *Agrobacterium tumefaciens* EHA105 harboring 35S:PgbZIP9 construct. These transformed seedlings were then put under salinity stress (250mM) for 12h. RNA was isolated and qRT-PCR was performed following the methods mentioned above.

For expression analysis of the *PgbZIP9* gene in callus, pearl millet seeds were germinated on callus induction media having MS media (Himedia), 0.3% phytagel (Hi-media), kinetin and 2,4-dichloro phenoxyacetic acid (2,4-D) at 25°C in dark condition following the protocol mentioned by Khirod K Sahoo et al. with modifications (Sahoo et al., 2011). Fully developed callus then sub-cultured on new callus induction for 7 days. These calluses were transformed with EHA105 harboring 35S:PgbZIP9 construct. Infected calluses were kept on co-cultivation media for 3 days. After this, the callus was subcultured on callus induction media for another 4 days, then transferred to salt stress media (250mM NaCl) for another 7 days. RNA samples were then extracted from control and salt-treated callus followed by qRT-PCR of the *PgbZIP9* gene, *ERD1, OST2* (Chakraborty et al., 2022) and *PgMyb88* (Chanwala et al., 2023b).

Also, expression profiling of the *PgbZIP9* gene was done from RNA samples extracted at different developmental stages of pearl millet, namely 2 weeks, 4 weeks, 8 weeks, pre-inflorescence, during-inflorescence, post-inflorescence and maturation.

### 2.15 Statistical analysis

All the experiments were done with three biological replicates and two technical of each replicates, and their mean values and respective standard deviations were used to plot graphs. Statistical significance between the mean values was determined using Student’s t-test and represented as asterisk sign ‘*’, where * p ≤ 0.05; ** p ≤ 0.01.

## 3 Results

### 3.1 Identification of bZIP TFs in pearl millet and sequence analysis

A total of 98 non-redundant bZIP proteins were identified through HMM search across the pearl millet genome as described in the method section. They were named PgbZIP1 to PgbZIP98 according to their specific location on the pearl millet chromosomes. Upon analyzing the physio-chemical features of identified PgbZIP proteins, we found that these proteins had varying amino acid lengths and molecular weight; the smallest protein was PgbZIP56, consisting of 69 amino acids and 7.925 kDa of weight, whereas PgbZIP69 was the largest one having 987 amino acids and 110.36 kDa of molecular weight. The predicted theoretical pI of PgbZIP proteins ranged between 4.2 (PgbZIP4) and 10.5 (PgbZIP31), whereas the low Grand average of hydropathicity (GRAVY) suggested that most of the PgbZIP proteins were hydrophilic and unstable. Also, we found that most PgbZIP proteins (74.48%) were predicted to be localized in the nucleus, indicating their possible role as transcription factor (**Supplementary Table 1**). We found 5 members of the identified PgbZIP proteins (PgbZIP2, PgbZIP6, PgbZIP7, PgbZIP79 and PgbZIP88) were predicted to contain transmembrane helices (**Supplementary Figure 1**).

### 3.2 Chromosomal mapping

**Figure 1:**
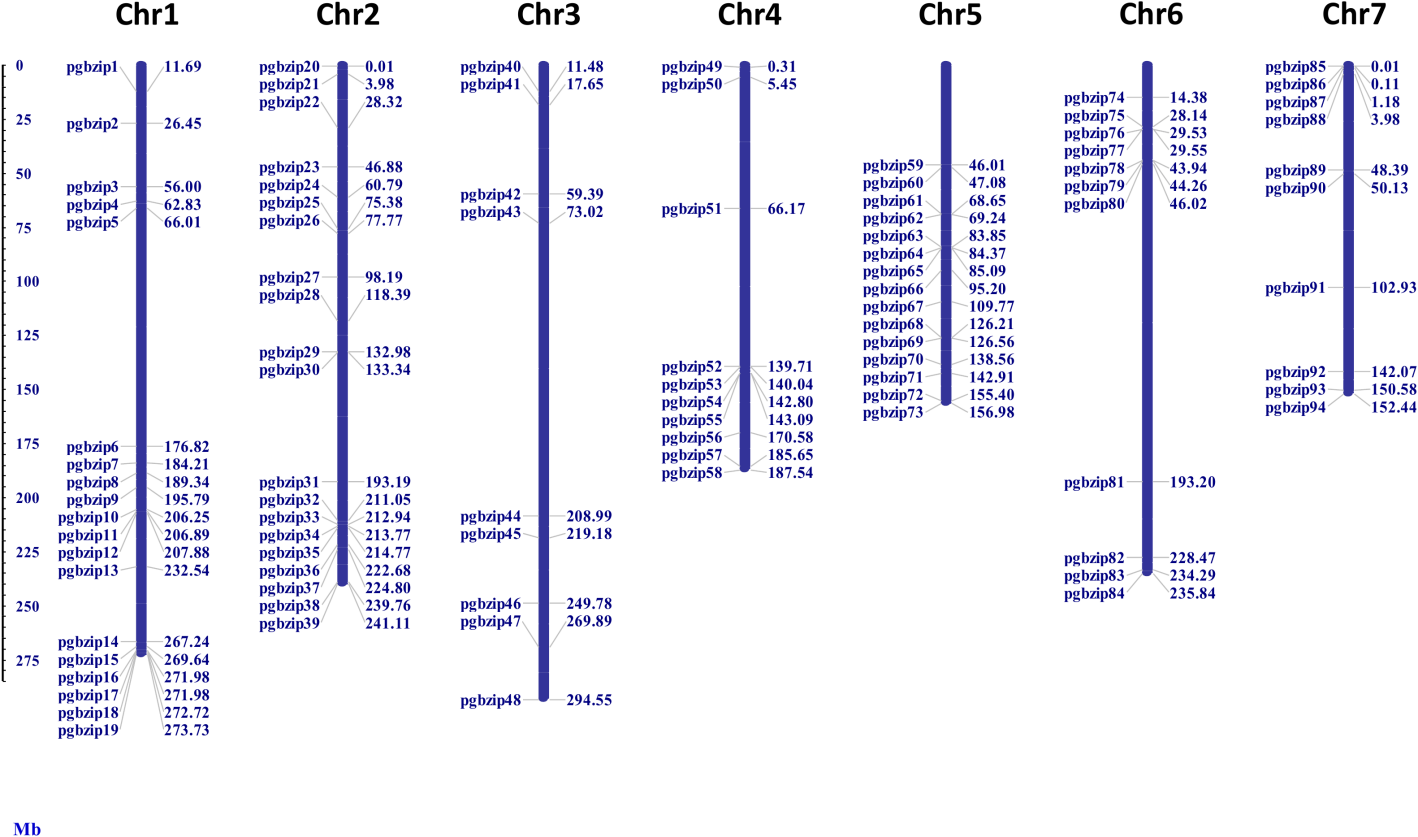
Location of *bZIP* family members on 7 chromosomes of pearl millet. The identified bZIP members were distributed on all the 7 chromosomes of pearl millet.

As shown in **Figure 1**, 94 out of 98 identified *PgbZIP* genes were physically mapped on the seven chromosomes of pearl millet with the help of MapInspect software. We found that they were distributed unevenly on pearl millet chromosome. While the remaining 4 PgbZIPs, namely *PgbZIP95, PgbZIP96, PgbZIP97* and *PgbZIP98* could not be mapped on the chromosomes due to unavailability of their chromosomal coordinates in pearl millet genome as they were found in the scaffold regions. Some of the pearl millet chromosomes had a higher number of *PgbZIP* genes, viz. 20 *PgbZIP* genes were located on chromosome 2, followed by 19 *bZIP* genes on chromosomes1, 15 *bZIP* genes on Chromosome 5, 11 *bZIP* genes on Chromosome 6, 10 *bZIP* genes each on Chromosome 4 and 7 and 9 *bZIP* genes were found on Chromosome 3.

### 3.3 Phylogeny, motif and gene structure analysis

**Figure 2:**
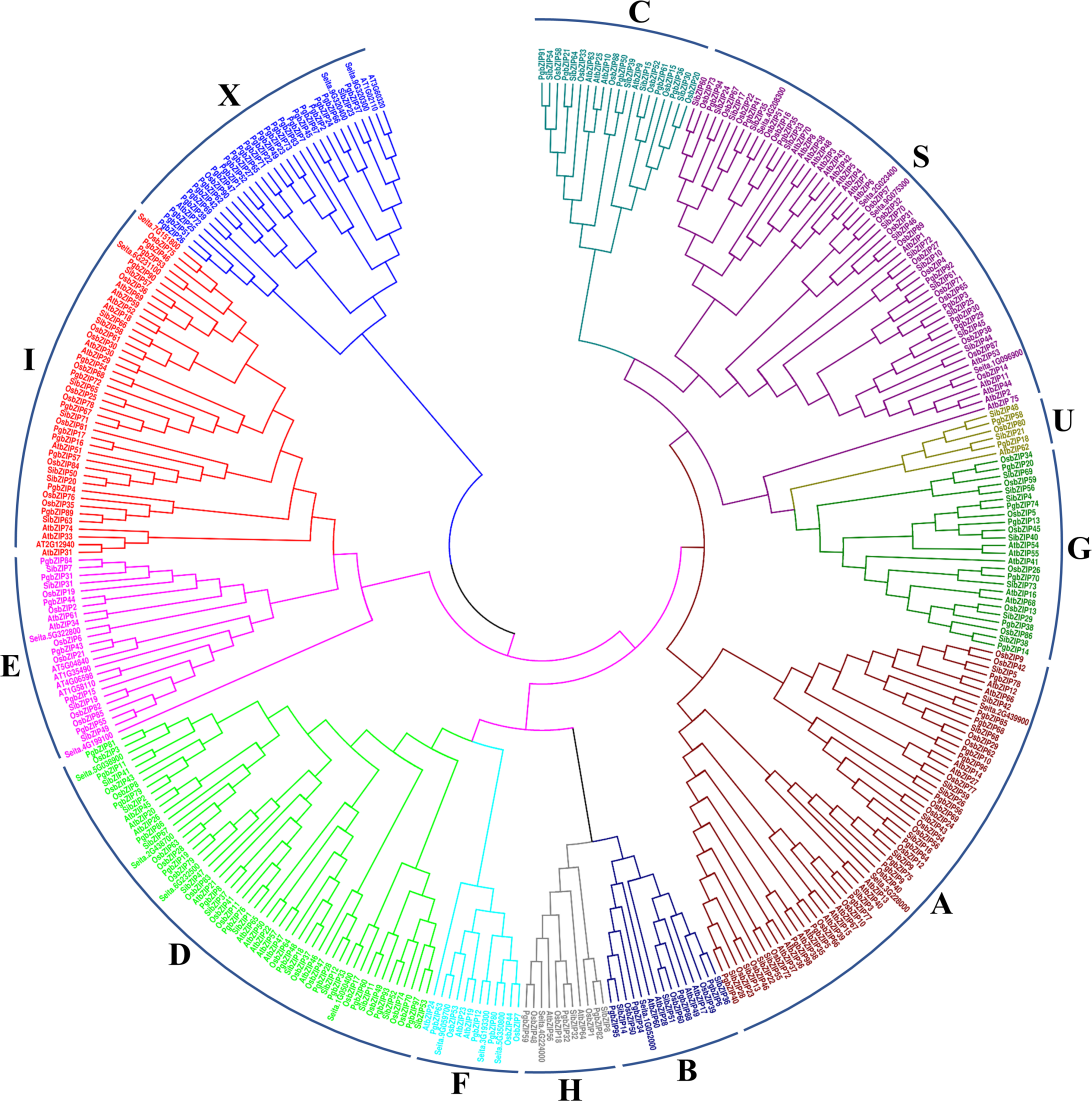
Phylogenetic tree of *P. glaucum* bZIP proteins with bZIP proteins of *A. thaliana, O. sativa, and S. italica.* A total of 356 proteins were aligned by MUSCLE, and a phylogenetic tree was constructed by MEGA v7.0 using the maximum likelihood method with 1000 bootstrap replication. The phylogeny classified the identified bZIP members into 12 groups.

For understanding the evolutionary progression of PgbZIP, an unrooted phylogenetic tree was constructed with the help of MEGA v7.0 as described in the method section. From phylogeny analysis, it was found that all the pearl millet bZIP proteins were divided into 12 groups (A-I, S, U and X). As shown in **Figure 2**, the highest number of PgbZIP proteins (25) belonged to the X-group, whereas A and D-groups had 13 members each. This is followed by 11 PgbZIP belonging to group I, 7 from the S-group, 6 from E-group, 5 from C-group, 4 from B-group, 3 each from F and H and 2 PgbZIP members from U-group.

**Figure 3:**
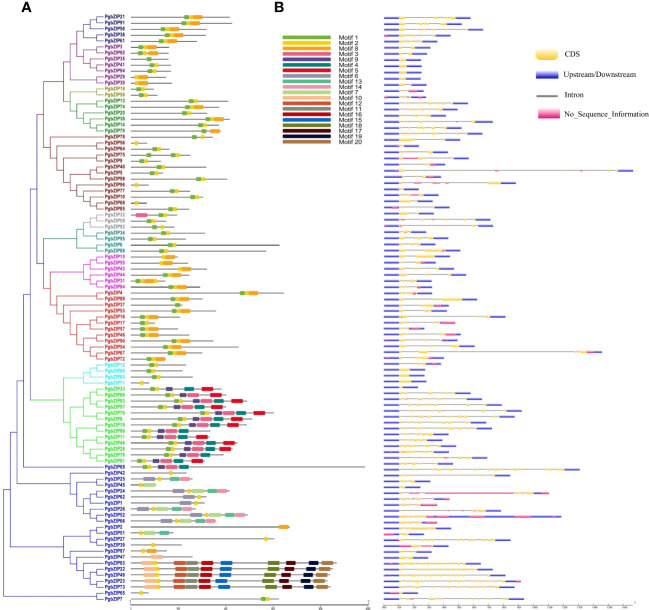
**(A)** Schematic representation of conserved motifs in PgbZIP proteins. Motifs are indicated by different color boxes for motifs 1–20, **(B)** Gene **s**tructure of the 98 putative pearl millet *bZIP* genes. Yellow boxes indicate exons, black lines indicate introns and blue boxes indicate upstream/downstream. Motif distribution and exon-intron arrangement was conserved among the groups.

Motif distribution is very crucial for the functionality of a protein, so we analyzed the conserved motifs in PgbZIP proteins through MEME suite. A total of 20 motifs (Motif 1 to Motif 20) were predicted across the PgbZIP protein members (**Supplementary Figure 2**). Motif 2 was found in almost all the PgbZIP proteins, whereas motif 1 was the second most abundant. Twelve PgbZIP proteins had only a single conserved motif (**Supplementary Table 2**). As shown in **Figure 3A**, motif distribution was also conserved among the phylogeny groups, majority of PgbZIP proteins of groups C, E and I contained motif 1, motif 2 and motif 8. Members of group-D PgbZIP proteins had motif 1 to motif 5 and motif 9. In group-X, the number of motifs was comparatively higher, such as PgbZIP83, PgbZIP22, PgbZIP49, PgbZIP23 and PgbZIP73 were exclusively consisting of motif 2, motif 10 to 12 and motif 15 to 20.

The gene structure (exon-intron distribution) analysis of *PgbZIP* genes was done with the GSDS server. We found that the exon-intron distribution was very much conserved among the phylogenetic groups (**Figure 3B**). All the *PgbZIP* gene members of group-C have six exons, group-U had 3-4 whereas group-F *PgbZIP* genes were intronless. Also, we observed *PgbZIP* genes of group-A had 2-9 exons; group-B had 2-3 exons; group-D had 7-12 exons; group-E members had 4-5 exons; group-G had 7-13 exons; group-H had 4-6 exons; group-I had 3-5 exons and group-S *PgbZIP* genes had 1-2 exons only. Interestingly we found that in unique group-X, the number of exons ranges from 1 (*PgbZIP65*) to 20 (*PgbZIP69*).

### 3.4 Gene duplication and synteny analysis

**Figure 4:**
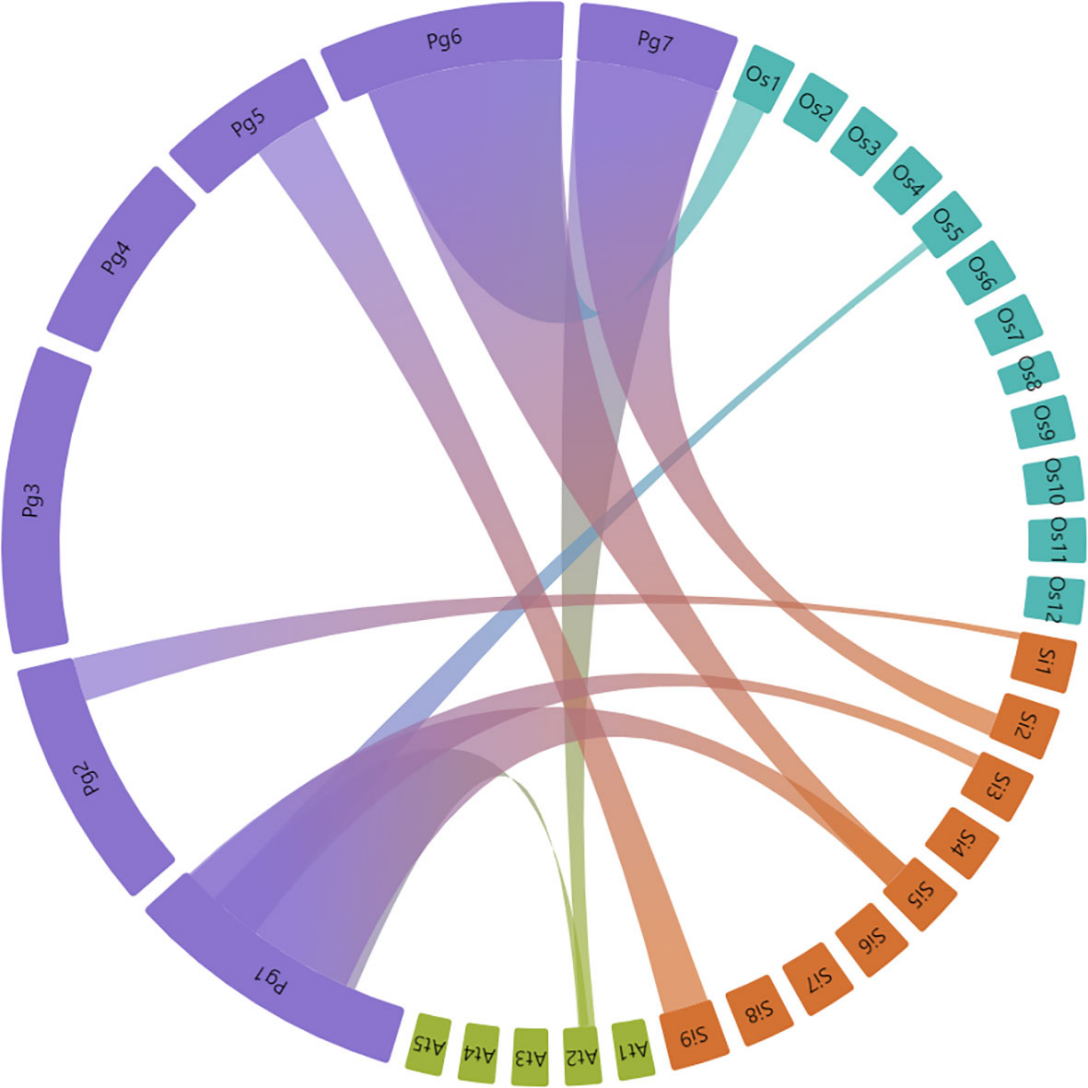
Synteny relationship between *P. glaucum*, *A. thaliana, O. sativa*, and *S. italica bZIP* genes. Each connected colored line shows duplicated gene pairs among the species. Closest relationship was observed between bZIP members of *P. glaucum* and *S. italica*.

Synteny analysis was done to determine the bZIP gene duplication frequency between pearl millet, *Arabidopsis*, rice and foxtail millet. Homologous *bZIP* genes including both orthologous and paralogous were predicted in the pearl millet. The *PgbZIP*s specifically from chromosome 1, were found to have an orthologous relationship with *Arabidopsis*, rice and foxtail millet (**Figure 4**). A total of 10 paralogous pairs, in the form of tandem duplication, were found in the *PgbZIP* genes. A total of 65 orthologous pairs were observed between the *PgbZIP* and bZIP family members of *Arabidopsis*, rice and foxtail millet (**Supplementary Table 3**). The highest collinear relationship was observed with foxtail millet having 73 (38.62%) collinear genes. This is followed by rice having 24 (12.83%) collinear genes and *Arabidopsis*, with 21 (11.86%) collinear genes. Also, we observed 9 collinear *PgbZIP* pairs both in rice and foxtail millet. Also, 20 collinear pairs were present only in foxtail millet, and 2 collinear genes were found in all reference species. Interestingly, 4 collinear pairs were only present in *Arabidopsis* but not in rice and foxtail millet. The non-synonymous mutation rate to synonymous mutation rate analysis showed that the Ka/Ks ratios of all collinear *PgbZIP* genes range between 0.0033 to 0.6343 (**Supplementary Table 4**).

### 3.5 Gene ontology annotation and cis-element analysis of *PgbZIP* genes

Gene ontology (GO) annotations analysis of identified PgbZIPs showed their potential role in biological processes, molecular functions, and cellular components. In the context of biological processes, PgbZIPs were found to be involved in the regulation of metabolic processes, cellular processes and different biological processes, as well as response to different stimuli, development processes, and reproductive processes (**Supplementary Figure 3**). Under molecular functions, PgbZIPs were predominantly linked with DNA-binding transcription regulator activity and DNA-binding. Within the cellular component category, GO term related to cellular anatomical entity was enriched and the majority of the annotated genes were found to be important components of the nucleus.

The bZIP TFs are stress-responsive genes that might be controlled under strong transcriptional regulation through their promoter region. To check this, we analyzed the 2000 bp upstream region of each the identified *PgbZIP* gene, considering them as potential promoter sequences of respective *bZIP* genes. Around 68 different cis-elements were predicted in the upstream region of *PgbZIP* genes, of them, major stress response-related cis-elements like Myb (90), STRE (95), MYC (83), as-1 (85), G-box (89), and ABRE (90) were highly abundant. Other notable cis-elements involved in important biological processes like biotic stress (AT-rich sequence, WUN motif), abiotic stress (DRE, ARE, LTR), physiological and developmental role (TCT-motif, Box-4, LAMP element, Sp1, GATA motif) were also predicted in the upstream region of *PgbZIP* genes (**Supplementary Figure 4**). The cis-element of core promoter regions (TATA- Box, CAAT- Box) were also found in upstream sequences of *PgbZIP* genes.

### 3.6 Protein-protein network analysis

The bZIP proteins have been reported to form homodimers or heterodimers for their DNA binding activity (Dröge-Laser et al., 2018). So we were interested to know whether members of PgbZIP proteins can interact with each other. As shown in **Supplementary Figure 5**, a protein-protein interaction network of PgbZIPs was predicted based on their orthologs in *Arabidopsis* and we observed multiple probable interactions. Proteins like VIP1 (PgbZIP89), bZIP17 (PgbZIP6, PgbZIP88), and bZIP24 (PgbZIP12) are associated with salt and osmotic responses. ABF1 (PgbZIP5), ABF2 (PgbZIP98), and ABI5 (PgbZIP9) are important proteins having a role in ABA-mediated stress responses. Their probable interaction suggests that PgbZIP proteins might interact to form protein complexes for mediating various biological functions.

### 3.7 Transcriptome analysis

To understand the expression pattern of *PgbZIP* genes under drought and salt stresses in pearl millet, we utilized the publicly available RNA-seq dataset. As shown in **Supplementary Figure 6**, *PgbZIP3, PgbZIP5, PgbZIP13, PgbZIP46, PgbZIP47, PgbZIP54, PgbZIP85, PgbZIP92* and *PgbZIP98* were differentially expressed under drought. Similarly, *PgbZIP3, PgbZIP5, PgbZIP9, PgbZIP46, PgbZIP47, PgbZIP54, PgbZIP61, PgbZIP85, PgbZIP92, PgbZIP97* and *PgbZIP98* members were found to have variable expression pattern under salinity stress.

### 3.8 Tissue-specific expression analysis

Based on *in-silico* features, genome-wide location and *in-silico* expression profile a total of 16 *PgbZIP* genes were selected for further expression analysis (**Supplementary Table 5**). To understand the tissue-specific expression of selected *PgbZIP* genes, we utilized the RNA samples extracted from the leaf, stem and root of pearl millet. Among the selected genes, most of the *PgbZIP* genes (*PgbZIP3, PgbZIP9, PgbZIP13, PgbZIP33, PgbZIP47, PgbZIP54, PgbZIP61, PgbZIP74, PgbZIP85, PgbZIP92, PgbZIP94* and *PgbZIP97*) had higher relative expression in the root of pearl millet while two *PgbZIP* genes (*PgbZIP5*, and *PgbZIP40*) showed higher expression in leaf and only single *PgbZIP* gene namely *PgbZIP46* showed higher mRNA level in stem samples (**Supplementary Figure 7**).

### 3.9 Expression analysis under abiotic stress and hormonal treatment

**Figure 5:**
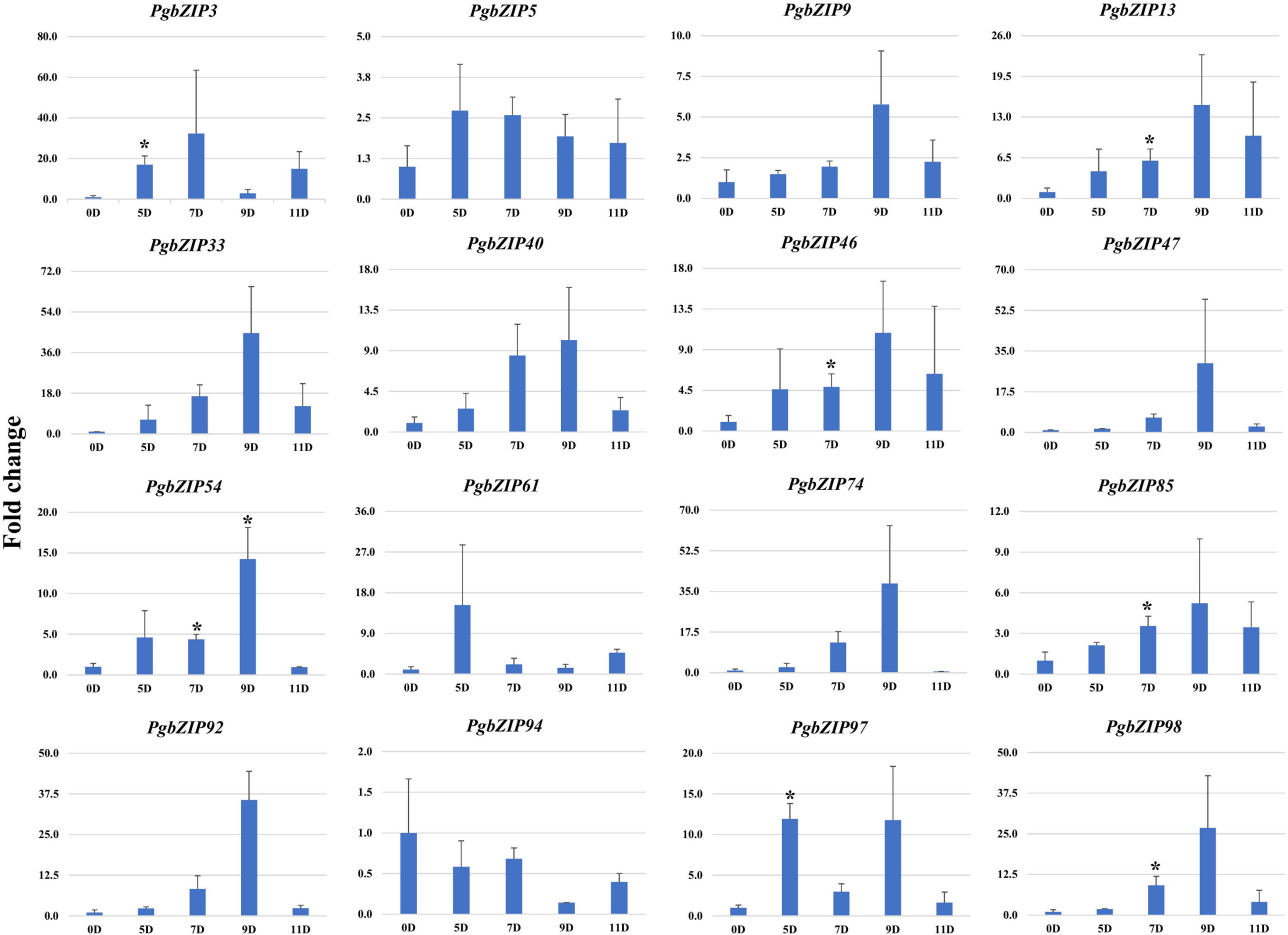
Expression analysis of *PgbZIP* genes under Drought stress at day 0, 5, 7, 9 and 11 post-treatment. Nearly All the candidate *PgbZIP* genes were upregulated under drought stress. The X-axis represents different time points and the Y-axis indicates relative expression level. A significant difference in the mean is indicated by *P < 0.05, as obtained by Student’s t-test.

**Figure 6:**
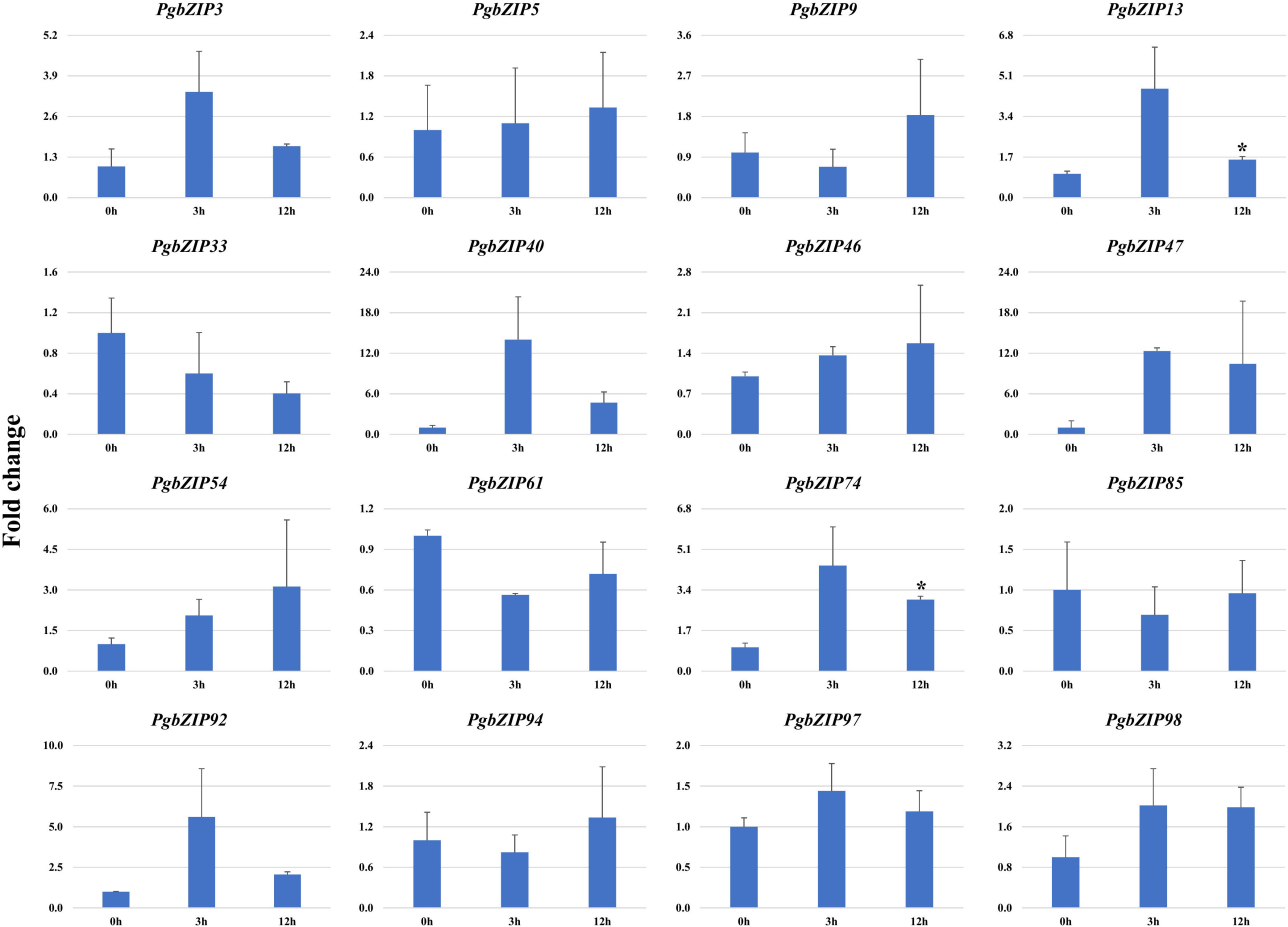
Expression analysis of *PgbZIP* genes under Heat stress at 0h, 3h and 24h post-treatment. Majority of *PgbZIP* genes were upregulated on heat application. The X-axis represents different time points and the Y-axis indicates relative expression level. The significant difference in the mean is indicated by *P < 0.05, as obtained by Student’s t-test.

**Figure 7:**
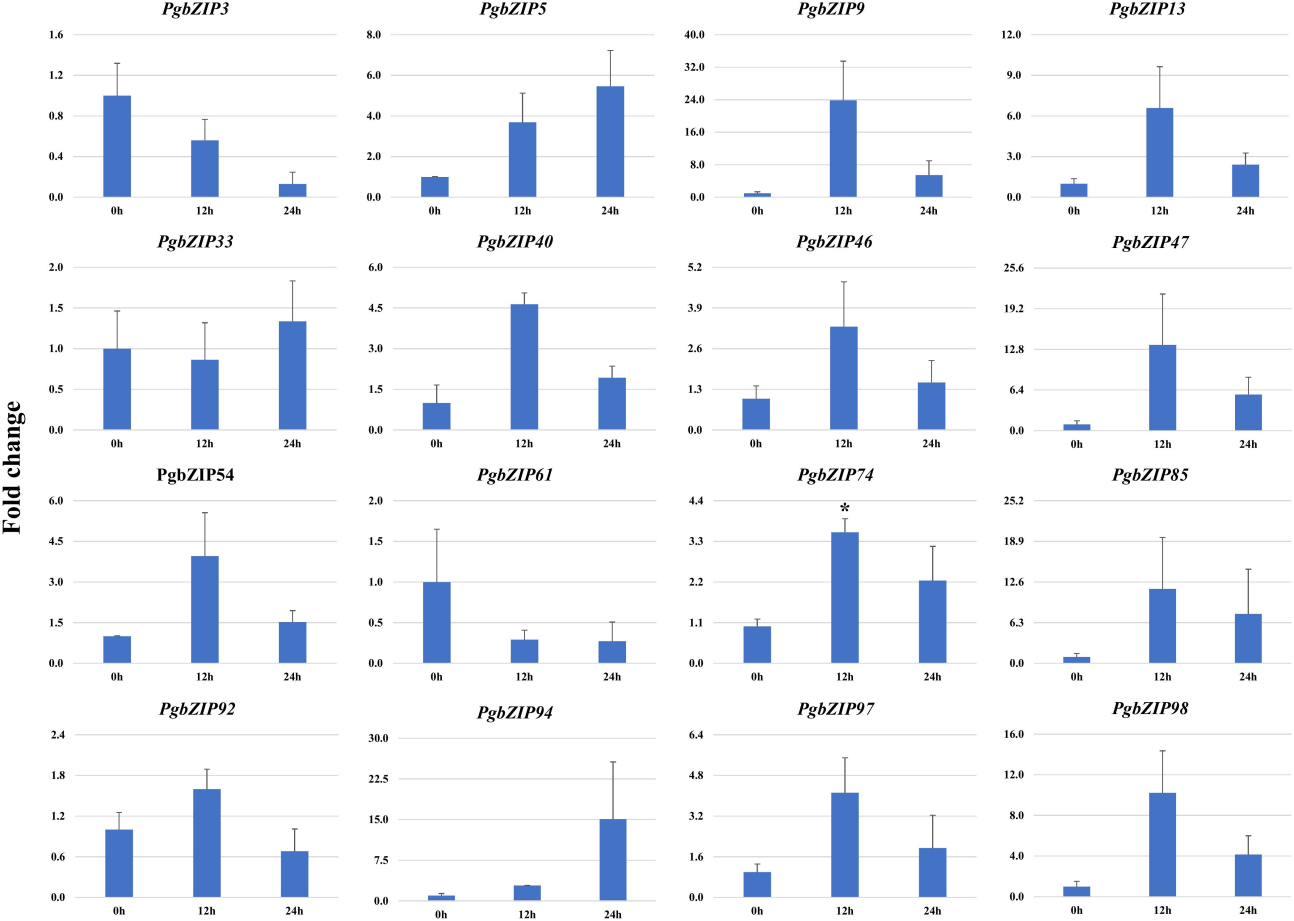
Expression analysis of *PgbZIP* genes under Salt stress at day 0h, 12h and 24h post-treatment. Majority of the *PgbZIP* genes were upregulated. The X-axis represents different time points and the Y-axis indicates relative expression level. A significant difference in the mean is indicated by *P < 0.05, as obtained by Student’s t-test.

To validate the role of *PgbZIP* genes in abiotic stress, we investigated the expression pattern of selected genes under different abiotic stresses (drought, heat and salinity). Upon induction of drought, all the candidate *PgbZIP* genes were found to be upregulated except *PgbZIP94* which was downregulated at all the time points. As shown in **Figure 5**, the majority of *PgbZIP* genes were highly expressed (upregulated) at all the stress induction time points but mRNA transcript levels of *PgbZIP9* (6 fold), *PgbZIP13* (15 fold), *PgbZIP33* (45 fold), *PgbZIP40* (10 fold), *PgbZIP46* (10 fold), *PgbZIP47* (30 fold)*, PgbZIP54* (15 fold), *PgbZIP74* (40 fold), *PgbZIP85* (5 fold), *PgbZIP92* (35 fold) and *PgbZIP98* (25 fold) were highest at 9^th^-day post drought stress. *PgbZIP3* showed 30-fold upregulation on the 7^th^ day of stress induction, whereas *PgbZIP5* and *PgbZIP61* showed early response having highest transcript accumulation on the 5^th^ day (**Figure 5**). On application of heat stress, majority of *PgbZIP* genes were upregulated of which five genes (*PgbZIP3, PgbZIP13, PgbZIP40, PgbZIP92* and *PgbZIP97*) showed early transcript accumulation and five genes (*PgbZIP46, PgbZIP47, PgbZIP54, PgbZIP74* and *PgbZIP98*) were upregulated at all the time points. While three *PgbZIP* namely *PgbZIP33, PgbZIP61*, and *PgbZIP85*genes were downregulated (**Figure 6**). Also on salt treatment, differential gene expression pattern was observed where most of the *PgbZIP* genes were upregulated. We found that *PgbZIP9* (24 fold), *PgbZIP13* (6 fold), *PgbZIP40* (4.5 fold)*, PgbZIP46* (3 fold)*, PgbZIP47* (13 fold)*, PgbZIP54* (4 fold)*, PgbZIP74* (3.5 fold)*, PgbZIP85*(10 fold)*, PgbZIP97* (4 fold)and *PgbZIP98* (10 fold) were upregulated at all the time points, but highest mRNA level was observed at 12-hour post stress induction. Two genes namely, *PgbZIP5* (5 fold) and *PgbZIP94* (15 fold) showed the highest transcript accumulation at 24-hour post-stress induction (**Figure 7**). Two genes, *PgbZIP3* and *PgbZIP61* were downregulated by 10-fold and 8-fold respectively upon salinity stress 24-hour post salt stress.

**Figure 8:**
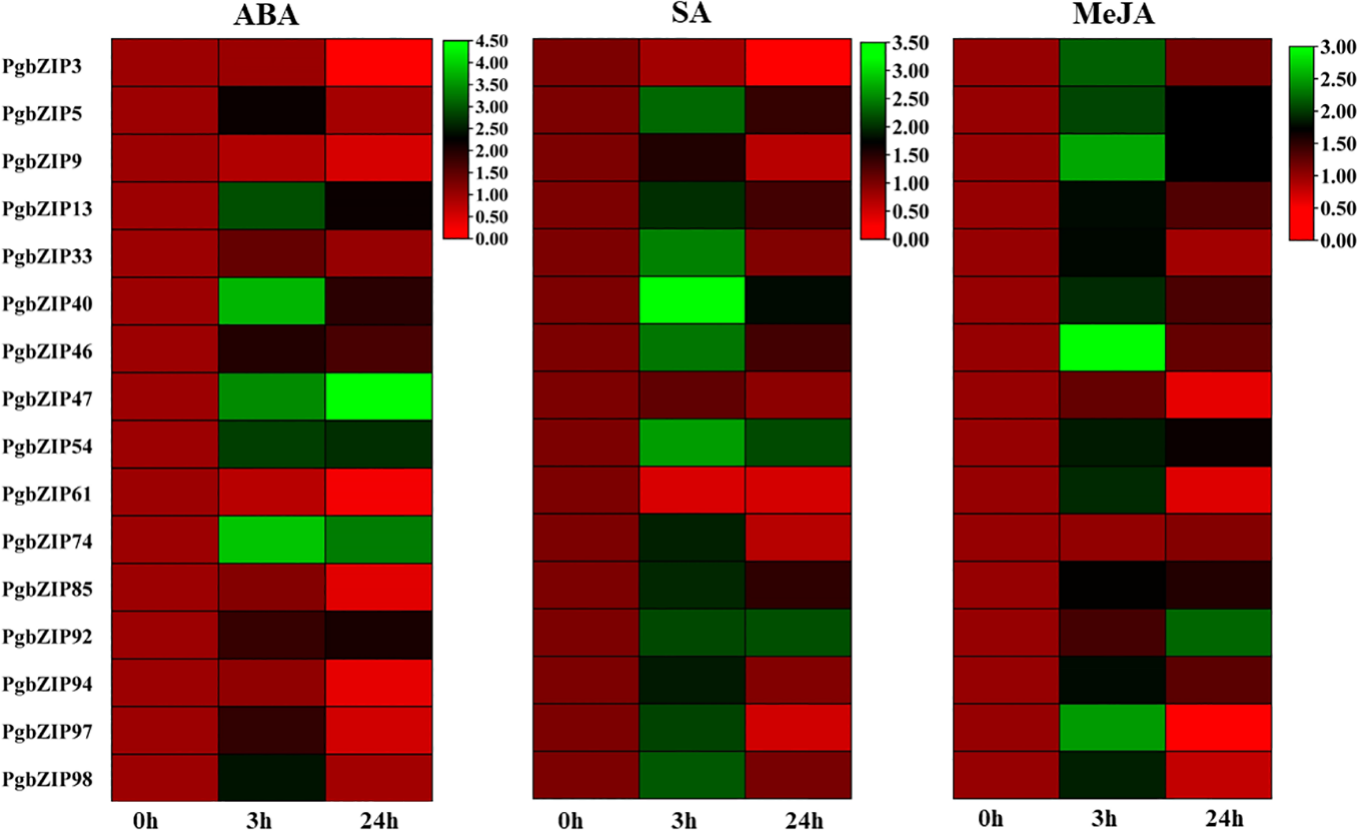
Heat map showing expression analysis of *PgbZIP* genes under SA, MeJA, and ABA hormonal stress at 0-hour, 3-hour, and 24-hour time points. The mRNA level has been normalized and the heat map was generated using TBtools v0.66831. The scale bar indicates lower (Red) to higher (Green) expressions level. The differential expression pattern was obtained for selected *PgbZIP* genes under all the phytohormonal treatments.

Phytohormones are very well-known for imparting stress response through their interplay with transcription factors. So we performed hormonal treatment to examine the expression profile of *PgbZIP* genes. As shown in **Figure 8**, we observed that majority of the candidate genes were induced upon all the 3 hormonal treatments (ABA, SA, and MeJA). Upon ABA treatment we found that most of the selected *PgbZIP* genes showed differential expression patterns. Of them seven genes were highly upregulated at early time point namely *PgbZIP5* (3.5 fold), *PgbZIP13* (6.5 fold), *PgbZIP33* (1.5 fold), *PgbZIP40* (12 fold), *PgbZIP46* (3 fold), *PgbZIP97* (2.5 fold)*, PgbZIP98* (4 fold). Five genes*, PgbZIP47*(7 fold)*, PgbZIP54* (2 fold)*, PgbZIP74*(8 fold) and *PgbZIP92* (3 fold) had higher mRNA levels at both 3 hours and 24 hours of treatment; while *PgbZIP3*, *PgbZIP9, PgbZIP61, PgbZIP85*, and *PgbZIP94* were highly downregulated by 6 fold, 2.5 fold, 5 fold, 3 fold and 3 fold respectively. Similarly, on SA treatment most of the *PgbZIP* genes were upregulated, *PgbZIP40, PgbZIP54, PgbZIP85* and *PgbZIP92* were highly upregulated at all the time points of SA treatment. *PgbZIP3* and *PgbZIP61* were significantly downregulated by 8 fold and 5 fold respectively. Most of the other candidate *PgbZIP* genes had higher transcript accumulation levels at early time point (**Figure 8**). Also, upon MeJA treatment almost all the candidate genes were upregulated, majority of them had higher expression at early time point (3h) while *PgbZIP5, PgbZIP9, PgbZIP54, PgbZIP85*, and *PgbZIP92* were upregulated at all the time points studied.

### 3.10 *PgbZIP9* encodes ABI5 type protein of bZIP family

**Figure 9:**
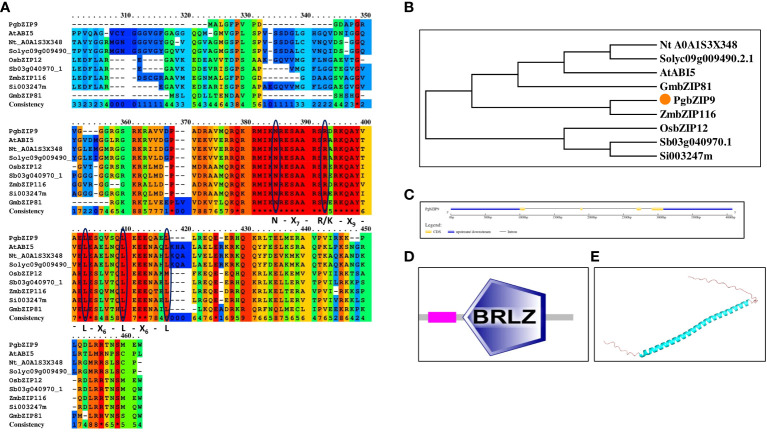
**(A)** Protein sequence alignment of PgbZIP9 protein along with bZIP proteins of other species confirmed the presence of bZIP domain, **(B)** Phylogenetic analysis of PgbZIP9 showed its closeness to *AtABI5*, **(C)** Exon-intron organization done with GSDS server, **(D)** bZIP domain confirmation with SMART server, **(E)** PgbZIP9 Structure prediction through Alpha fold server showed disordered N-terminal and C-terminal regions.

In the arid and semi-arid regions, salinity is a major problem; pearl millet being grown in these regions are frequently exposed to such salinity stress (Krishnamurthy et al., 2007). So, we selected PgbZIP9, having the highest expression level under salinity stress for further analysis to look into its potential role in salinity stress tolerance. The isolated *PgbZIP9* had an open reading frame of 116 amino acids, which is ten amino acids less (with an intact start codon, ATG and stop codon, TAG) than the reference sequence (Pgl_GLEAN_10009291). Sequence analysis showed that isolated PgbZIP had characteristic N-X_7_-R-X_9_-L-X_6_-L-X_6_-L amino acid region (**Figure 9A**). The predicted molecular weight of PgbZIP9 was around 13.39 kDa and its theoretical pI was 10.16. The phylogenetic analysis along with sequence analysis suggested the close relationship of PgbZIP9 with the ABI5 type of bZIP proteins of other species (**Figure 9B**). Gene structure analysis showed that the *PgbZIP9* gene consists of four exons and three introns (**Figure 9C**). As shown in **Figure 9D** putative PgbZIP9 protein contained typical bZIP domain (amino acid 33 to 96). Also, we predicted its structure through the Alpha fold which showed that only the central bZIP domain forms the α-helical structure whereas the N-terminal and C-terminal regions are highly disordered portions (**Figure 9E**).

### 3.11 PgbZIP9 is a nuclear-localized protein

**Figure 10:**
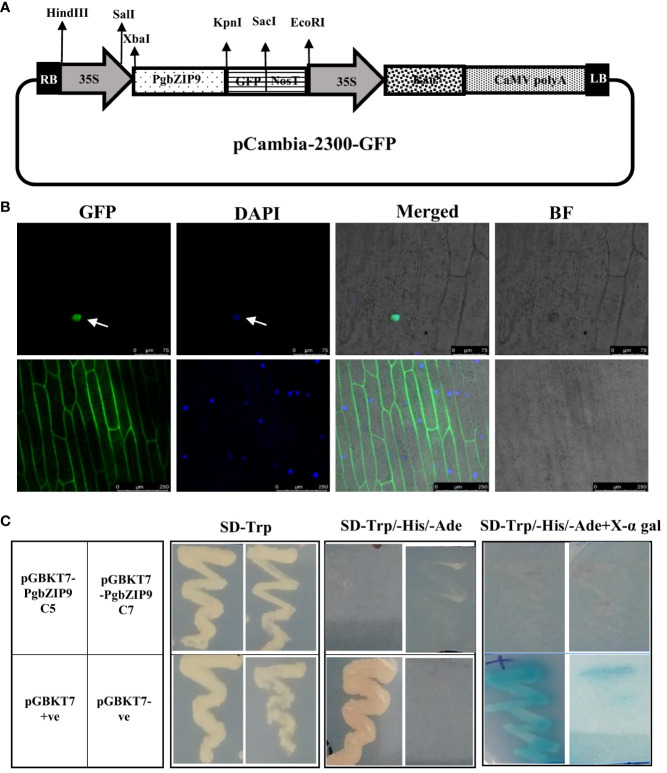
Subcellular localization and transactivation activity analysis of PgbZIP9. **(A)** Schematic representation of 35S:PgbZIP9-GFP construct. **(B)** Confocal image of subcellular location of *PgbZIP9* gene along with vector control. GFP, Green fluorescence; DAPI, 4’,6-diamidino-2-phenylindole; Merged, Merged image of GFP; DAPI and Bright field (BF). *PgbZIP9* was a nuclear protein. **(C)** Transcriptional activation assay of PgbZIP9 in a yeast expression system. No transcriptional activity was observed in yeast.

The PgbZIP9 protein contained a nuclear localization signal, and it was predicted to be localized in the nucleus through the WolfPsort server. Further, we performed the sub-cellular localization assay by particle bombardment of the onion epidermal cells with GFP-fused PgbZIP9 construct derived under the CaMV35S promoter (**Figure 10A**). The CaMV35S:GFP construct was taken as control. Under the confocal microscope, we observed that an intense green fluorescence signal was visualized specifically from the nucleus in case of CaMV35S:PgbZIP9:GFP fusion protein, whereas in CaMV35S:GFP construct green fluorescence signal was visible from most of the cell (**Figure 10B**). This confirmed the nuclear localization of PgbZIP9 protein.

The yeast two-hybrid system from Takara was utilized to study the transcriptional activation ability of PgbZIP9. As shown in **Figure 10C**, all transformed yeast cells grew well on the SD/-Trp medium. The positive control (pGBKT7) grew well on SD/-Trp/-His/-Ade media and gave blue on the SD/-Trp/-His/-Ade/X-a-gal medium, but the negative control and pGBKT7-PgbZIP9 transformant did not grow on the same medium. This result indicated the inability of the *PgbZIP9* gene to transactivate the reporter genes in the yeast system.

### 3.12 *PgbZIP9* expression analysis

For understanding the expression pattern of *PgbZIP9* and its role in stress responses, we transiently overexpressed the *PgbZIP9 gene* in the 7-day pearl millet seedlings and pearl millet callus. We observed that the transient overexpression of *PgbZIP9* led to its higher transcript accumulation in transformed samples compared to non-transformed (wild-type). Also, under salinity stress the expression level of *PgbZIP9* was enhanced upto 10 fold in transformed pearl millet seedlings and callus compared to the untreated transformed samples (**Supplementary Figures 8A, B**). The expression levels of some of the reported stress genes of pearl millet, namely *ERD1, OST2* and *PgMYB88* were upregulated under salt stress in transformed samples, which further suggests the probable role of the *PgbZIP*9 gene under salt stress.

To investigate the importance of *PgbZIP9* genes in the growth and development of pearl millet, we analyzed its expression at different growth stages. We found that it had higher expression in the early period of pearl millet growth (2 weeks and 4 weeks). The expression level was reduced before and during the inflorescence while its expression further increased (slightly) post-inflorescence (**Supplementary Figure 8C**).

## 4 Discussion

Pearl millet is a highly important crop that has a natural ability to survive in harsh marginal lands. The whole genome sequencing of pearl millet has opened the door to understand the mechanistic view behind their stress responses (Varshney et al., 2017). Since then, few stress-related transcription factors namely WRKY, NAC, GRAS, MYB, and DoF have been studied (Chanwala et al., 2020; Dudhate et al., 2021; Jha et al., 2021; Qu et al., 2023; Chanwala et al., 2023a, b). However, the *bZIP* gene family in pearl millet has not been explored yet. The bZIP transcription factors are abundant in the plant system and play crucial roles in plant growth, development, phytohormonal signaling, metabolism, and stress responses (Nijhawan et al., 2008; Dröge-Laser et al., 2018). Here, we have identified 98 members of the bZIP TF gene family through a genomic survey of pearl millet. We observed the number of identified bZIP TFs in pearl millet is higher compared to *Arabidopsis thaliana* (78), *Oryza sativa* (89), *Sorghum bicolor* (92), *Solanum lycopersicum* (69), *Vitis vinifera* (55), *Phaseolus vulgaris* (84), *Solanum tuberosum* (65) but lesser than *Malus domestica* (116), *Zea mays* (125), *Glycine max* (131), and *Triticum aestivum* (227) (Liao et al., 2008; Nijhawan et al., 2008; Wang et al., 2011; Wei et al., 2012; Dröge-Laser et al., 2018; Zhang et al., 2021; Liang et al., 2022). This variation in *bZIP* numbers across species might be gene duplication or loss during the evolution.

Membrane-bound transcription factors are quite prevalent and account for around 10% of total transcription factors in plants. However, they are presumed to be present in a dormant state and get functionally active only upon proteolytic cleavage or ubiquitin-proteasome pathway (Seo et al., 2008). Very few bZIP TFs have been reported as membrane proteins like AtbZIP17, AtbZIP28 and AtbZIP60 (Iwata and Koizumi, 2005; Liu et al., 2007a, b). Our study has predicted five membrane-bound PgbZIP proteins (PgbZIP2, PgbZIP6, PgbZIP7, PgbZIP79 and PgbZIP88). They all have a single transmembrane helix extending from inside to outside the cell membrane. Disproportionate distribution of PgbZIP members was observed on the chromosomes of pearl millet. Chromosomes 1 and 2 can be considered as the hotspot regions for *PgbZIP* genes and distribution may lead to species differentiation.

All the PgbZIP proteins were categorized into 12 groups based on phylogeny analysis which was nearly identical to *Arabidopsis*, rice and foxtail millet bZIP protein classification (Nijhawan et al., 2008; Baoling et al., 2016; Dröge-Laser et al., 2018). Each of the phylogenetic groups corresponds to the specific function of the plant. The number of PgbZIP proteins in groups A and D were similar to *Arabidopsis*, indicating their identical role in pearl millet physiology. However, comparatively fewer *PgbZIP* genes were found in the S group which might be because of gene loss or they differentiated into another group in pearl millet during evolution (Fedorova and Fedorov, 2003). Interestingly, many *PgbZIP* genes were clustered in a particular clade (group X), suggesting their specific involvement in pearl millet physiology. Motif distribution and gene structure features were conserved among the groups. The unique X-group members were observed to have maximum numbers of motifs as well as introns in pearl millet.

Gene duplication and gene loss could be the driving forces for functional variation and gene family expansion (Sampedro et al., 2005). Duplication events were higher between *S. italic* and *P. glaucam* compared to *O. sativa* and *A. thaliana*. Tandem and segmental duplication have been reported to have a great contribution to gene family expansion (Vision et al., 2000; Cannon et al., 2004). In this study, only 10 tandem duplication events were predicted but no segmental duplications. Foxtail millet was predicted to share the highest collinear genes around 38.62% compared to other reference species. Also, 9 collinear pairs of *PgbZIP* genes were found in foxtail millet and rice but not in *Arabidopsis* which indicated that they probably formed after the bifurcation of dicot and monocot species. Similarly, 4 *PgbZIP* collinear genes were found only in *Arabidopsis* suggesting their disappearance during the dicot and monocot branching. Interestingly, 20 *PgbZIP* collinear genes were exclusively present in foxtail millet which propounds their possible formation during millet evolution. These findings show the evolutionary progression of *PgbZIP* family genes in pearl millet. Ks/Ka values are important for understanding genomic evolution and to know the selection pressure during the course of evolution (Hurst, 2002). Our study suggests that all the bZIP genes in pearl millet had a purifying selection; their Ks/Ka values were less than 1 (**Supplementary Table 4**).

Transcription factors regulates the expression of downstream genes by binding with its cognate cis-elements to mediate the stress response (Liu et al., 2020). The availability of specific cis-elements on the gene promoter region dictate their possible functions. A wide range of cis-elements associated with plant growth and development, stresses, and signaling were on the upstream region of *PgbZIP* genes which suggest their diverse role in the plant system (Zhang et al., 2021). Specifically major abiotic stress associated cis-elements like W-box, MYB, STRE, ABRE, as-1, G-box and MYC were present which further indicates the involvement of *PgbZIP* genes in abiotic stress responses which was also evident in earlier studies (Sornaraj et al., 2016; Khadanga et al., 2022; Sherpa et al., 2023). Protein-protein interaction analysis showed multiple interactions between orthologous bZIP proteins in Arabidopsis, indicating probable interaction among PgbZIP protein. These predicted interaction needs to be further verified in pearl millet.

The bZIP transcription factors have an important role in plant growth and development processes. The *PgbZIP* genes of different groups are linked to these roles like group A-bZIP gene, ABI5, controls the plant floral transition by regulating flowering locus C (Shu et al., 2018). *TGA8*, a group D-bZIP member, has been reported to control floral organ formation (Maier et al., 2011). Group H-bZIP members were specifically related to various developmental processes like hypocotyl and lateral growth (Gangappa and Botto, 2016; Van Gelderen et al., 2018). GBF1, a G-group protein has been found to promote lateral root development (Maurya et al., 2015). Expression patterns in tissues of *PgbZIP* genes could hint at their role in growth and developmental functions. The *PgbZIP13* and *PgbZIP74* genes, which belong to group G had very high expression in roots which correlates their function to group G *Arabidopsis bZIP* genes. Similarly, S-group bZIP genes namely *PgbZIP3, PgbZIP92* and *PgbZIP94* showed very high expression in root which were in good co-relation with *Arabidopsis* S-group protein *bZIP11* that has been reported to control primary root growth (Hartmann et al., 2015).

To understand the expression pattern of *PgbZIP* genes under abiotic stress, transcriptome analysis showed that the majority of *PgbZIP* genes were differentially expressed. To further look into the role of *PgbZIP* genes in stress, we studied the *PgbZIP* members of various groups. In *Arabidopsis*, bZIP members of most of the groups have been reported to take part in stress response (Dröge-Laser et al., 2018). Similarly, the selected candidate *PgbZIP* genes from different groups have been found to play a critical role in abiotic stress. They showed different expression patterns in response to drought, heat and salt stresses. We found that *PgbZIP13, PgbZIP40, PgbZIP46, PgbZIP47, PgbZIP54, PgbZIP74, PgbZIP92, PgbZIP97*, and *PgbZIP98* were upregulated under all the three stresses (drought, heat and salt) suggesting their high importance in stress tolerance in pearl millet. Almost all the candidate genes were upregulated upon drought stress in pearl millet, a similar result was observed in wheat (Liang et al., 2022). Specifically, the expression of *PgbZIP74* and *PgbZIP92* increased by 30-fold under drought stress. Upon salt stress, *PgbZIP9* showed around 25-fold higher expression. Also, *PgbZIP61* was observed to be downregulated upon drought, heat and salt stress whereas *PgbZIP94* was negatively regulated under drought and heat stress. The phylogenetic tree analysis further emphasizes the association of PgbZIP genes in stress response. Recent studies have shown that members of group A *bZIP* genes like *OsbZIP23, OsABI5, OsABF1* and *AtbZIP35-AtbZIP39* were involved in imparting stress response (Liang et al., 2016). The pearl millet *bZIP* genes like *PgbZIP5, PgbZIP9, PgbZIP40* and *PgbZIP98* belonged to A-group and had differential expression under stress. A wheat *bZIP* gene, *TabZIP19* (G-group) had higher expression under stress; similarly, *PgbZIP13* and *PgbZIP74* (group G members) showed very high expression under different stresses (Liang et al., 2022). In *Arabidopsis*, *bZIP1* a S-group gene responded to salt stress, also in pearl millet *PgbZIP92* and *PgbZIP94* (S-group) showed upregulation upon salt stress (Dröge-Laser et al., 2018).

Phytohormones are essential for plants to adapt the harsh environmental conditions (Verma et al., 2016). They crosstalk with transcription factors to mediate the stress responses. So, to explore the connection between *PgbZIP* genes and phytohormones, we analyzed the expression profile of selected *PgbZIP* genes under hormonal treatments (ABA, SA and MeJA). Various studies indicate that bZIP TFs impart stress tolerance through ABA signaling in *Arabidopsis*, rice and tomato (Banerjee and Roychoudhury, 2017). In this study, we also observed that *PgbZIP* genes (*PgbZIP40, PgbZIP46, PgbZIP47, PgbZIP54, PgbZIP74, PgbZIP92, PgbZIP97*, and *PgbZIP98)* which showed elevated expression under abiotic stresses and had higher transcript level on ABA treatment. The promoter region of these *PgbZIP* genes contained multiple numbers of ABA-responsive cis-element (ABRE). Also, candidate *PgbZIP* genes showed nearly the same expression pattern upon salicylic acid treatment. This suggests that *PgbZIP* genes probably induce the abiotic stress response in pearl millet through the synergistic action of ABA and SA. This type of synergistic role of ABA and SA has been reported earlier (Jesus et al., 2015; Wang et al., 2018). *PgbZIP54* and *PgbZIP92* were upregulated under all the hormonal treatments, whereas *PgbZIP98* was upregulated at an early time point (3h). *PgbZIP61* was downregulated under ABA and SA. This expression pattern of *PgbZIP* genes gives insight into their possible regulatory function in phytohormonal pathways in pearl millet.

To further investigate the effect of *bZIP* genes in salinity stress in pearl millet, we isolated and cloned *PgbZIP9*, which was predicted as homologous of AtABI5. PgbZIP9 (*PgABI5*) had characteristic bZIP domain. Subcellular localization assay confirmed its location in the nucleus which is in accordance with the biological function of transcription factors; however, it did not show transactivation feature in yeast. Transient overexpression of *PgbZIP9* in pearl millet seedlings and callus showed a very high transcript level of *PgbZIP9* upon salt stress. Very little information is available about the stress-responsive genes in pearl millet; we used two genes, *ERD1* and *OST2*, which were earlier reported (Chakraborty et al., 2022). Under salt stress, early responsive dehydration 1 (*ERD1*) has been reported to have upregulation in multiple studies (Liu et al., 2022). Similarly, Open Stomata 2 (*OST2*) which encodes a plasma membrane H^+^ATPase is crucial for ion homeostasis during salinity stress (Zhang et al., 2022). Earlier, *PgMyb88* was also proposed to have a role under salt stress in pearl millet (Chanwala et al., 2023b). These genes viz. *ERD1*, *OST2* and *PgMyb88* were upregulated under salt stress in transformed samples (both seedlings and callus). Upregulation of these genes upon salt stress in transiently overexpressed pearl millet seedlings and callus emphasizes the importance of the *PgbZIP9* gene in salt stress tolerance. However, these data are very primitive and need further characterization. Also, the higher expression of *PgbZIP9* genes in 2-week and 4-week-old pearl millet suggests its role in early stages of pearl millet growth and development.

Altogether, under both abiotic stress and hormonal treatment majority of *PgbZIP* genes were induced which emphasizes their systematized action in pearl millet. Interestingly, multiple numbers of *PgbZIP* genes were positively induced upon all the treatments, this signifies the importance of *bZIP* genes in pearl millet stress tolerance. These findings established the gravity of *PgbZIP* genes in abiotic stress response through phytohormonal pathways. However, this study is the nascent work about possible involvement of bZIP TFs in pearl millet under stress response and needs further investigation to understand their role in imparting resistance to pearl millet under stress conditions through gene silencing/overexpressing (gene editing approaches).

## 5 Conclusion

This study identified 98 bZIP Tfs in the pearl millet genome through the genomic survey. These identified *bZIP* genes were divided into 12 groups based on phylogeny analysis; motif distribution and structural features were conserved among the groups. Gene duplication events were observed, and Ks/Ka analysis suggested the evolutionary progression of *PgbZIP* genes under purifying selection. Transcriptome analysis indicated the differential expression of PgbZIP genes under abiotic stress, which was supported by the presence of various abiotic stress-responsive cis-elements. Interestingly, we found that multiple pearl millet *bZIP* genes (*PgbZIP40, PgbZIP47, PgbZIP74*, and *PgbZIP97*) were highly upregulated at either time point under all the abiotic stresses and phytohormonal treatments, whereas *PgbZIP61* was downregulated under all the stresses except MeJA treatment. Further, we demonstrated that PgbZIP9 (PgABI5) a nuclear-localized protein induced the stress-responsive genes under salinity stress. These results laid a strong foundation for future works to dissect the molecular mechanism and downstream pathway analysis of bZIP transcription factors in stress responses in pearl millet. Further candidate *PgbZIP* genes can be employed to develop climate-resilient crop plants.

## Data availability statement

The original contributions presented in the study are included in the article/**Supplementary Material**. Further inquiries can be directed to the corresponding author.

## Author contributions

DJ: Data curation, Formal analysis, Investigation, Methodology, Software, Validation, Writing – original draft, Writing – review & editing. JC: Data curation, Formal analysis, Investigation, Methodology, Software, Validation, Writing – original draft, Writing – review & editing. PB: Data curation, Formal analysis, Software, Writing – original draft. ND: Conceptualization, Funding acquisition, Investigation, Project administration, Supervision, Validation, Writing – review & editing.

## Supplementary material

Supplementary Figure 1 Membrane-bound bZIP transcription factors with a transmembrane motif. 5 member of PgbZIP were predicted to have transmembrane region.

Supplementary Figure 2 Representative logo of the predicted motif (Motif 1- Motif 20) of PgbZIP proteins.

Supplementary Figure 3 The Gene Ontology (GO) analysis defining the biological processes, molecular function and cellular components of PgbZIP proteins by Level (2).

Supplementary Figure 4 Cis-element analysis of *PgbZIP* genes in 2000 bp upstream region. Color of the bars indicates the group (shown in legend) it belongs. Diverse cis-elements were predicted on the upstream region of *PgbZIP* genes.

Supplementary Figure 5 Protein-protein interaction network for PgbZIPs based on their orthologs in Arabidopsis. The PgbZIP proteins were shown in the red font below with the Arabidopsis orthologs. The PgbZIP proteins were predicted to have interaction with each other.

Supplementary Figure 6
*In-silico* expression analysis of *PgbZIP* genes under drought and salt stress using publicly available RNA-seq data. The scale bar indicates lower (Red, -4) to higher (Green, 3) expressions level. Differential expression pattern was obtained for *PgbZIP* genes,

Supplementary Figure 7 Tissue-specific expression analysis of *PgbZIP* genes in Leaves, Stem and Root tissues of pearl millet. Most of the *bZIP* genes were highly expressed in the root. The significant difference in the mean is indicated by *P < 0.05, **P < 0.01, as obtained by Student’s t-test.

Supplementary Figure 8
**(A).** Expression level of *PgbZIP9* and stress-related genes in transiently overexpressed pearl millet seedlings. Overexpressed seedlings showed higher expression level of *PgbZIP9* and stress genes. Wt: Wild type pearl millet seedlings, Tr: Pearl millet seedlings transiently transformed with *PgbZIP9* gene **(B).** The expression level of *PgbZIP9* and stress-related genes in transiently overexpressed pearl millet callus. Overexpressed callus showed higher expression level of *PgbZIP9* and stress genes. Wt: Wild type pearl millet callus, Tr: Pearl millet callus transformed with *PgbZIP9* gene (without stress), ST: Transformed callus treated with 250mM NaCl **(C).** Expression analysis of *PgbZIP9* at different growth of pearl millet including 2-week (2W), 4-week (4W), 8-week (8W), pre-inflorescence (PrI), during-inflorescence (DI), post-inflorescence (PoI) and maturation (M). *PgbZIP9* had higher expression level at early stage of life cycle in pearl millet.
