# Supplementary figures and images for: “Genome-wide identification of bZIP gene family in Pearl millet and transcriptional profiling under abiotic stress, phytohormonal treatments; and functional characterization of PgbZIP9”
Source: Front Plant Sci. 2024 Feb 26;15:1352040. doi: 10.3389/fpls.2024.1352040 (PMC10925649; doi:10.3389/fpls.2024.1352040)

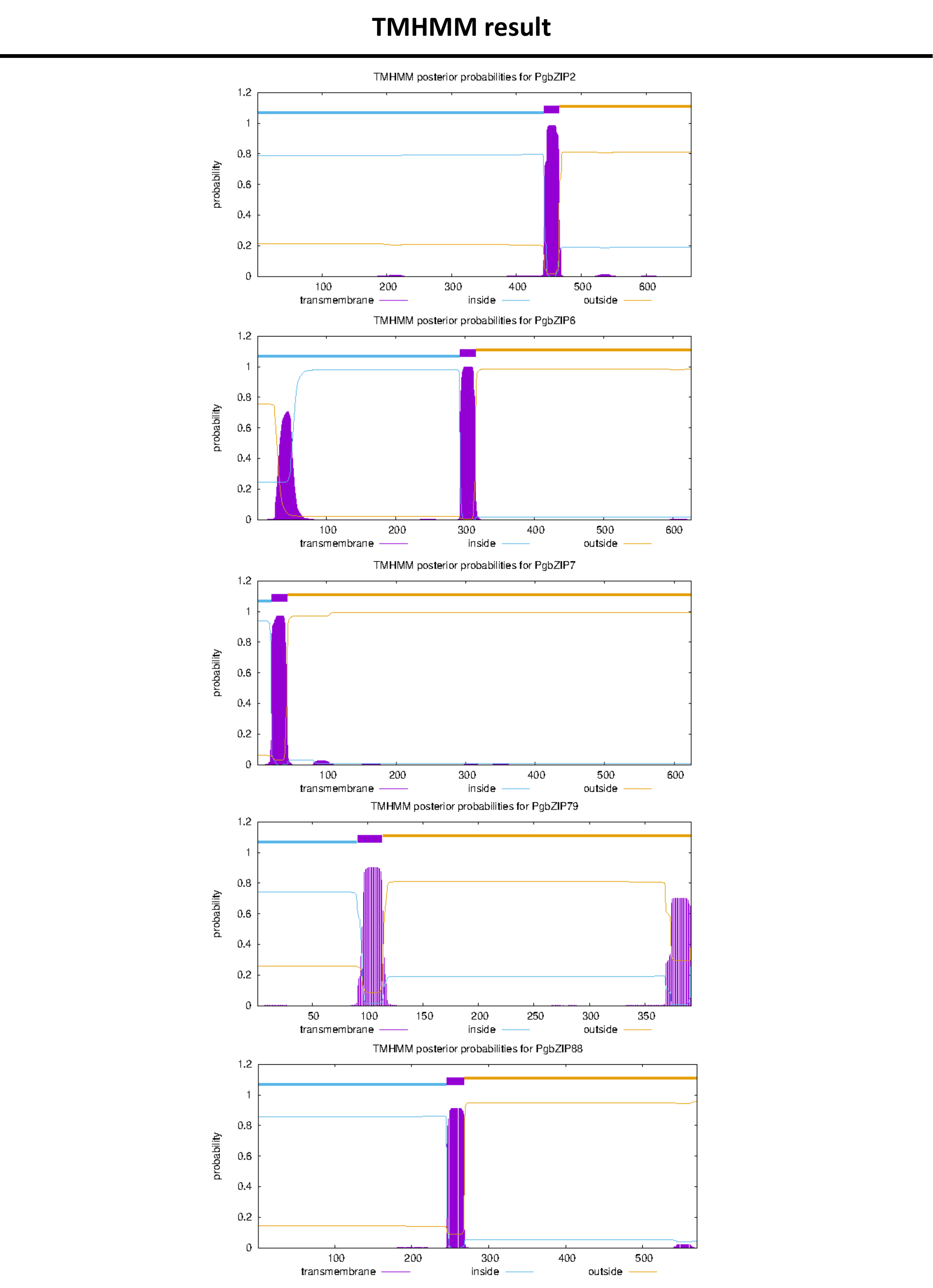

Supplement: Supplementary Figure 1 — Membrane-bound bZIP transcription factors with a transmembrane motif. 5 member of PgbZIP were predicted to have transmembrane region. [file Image_1.tiff]

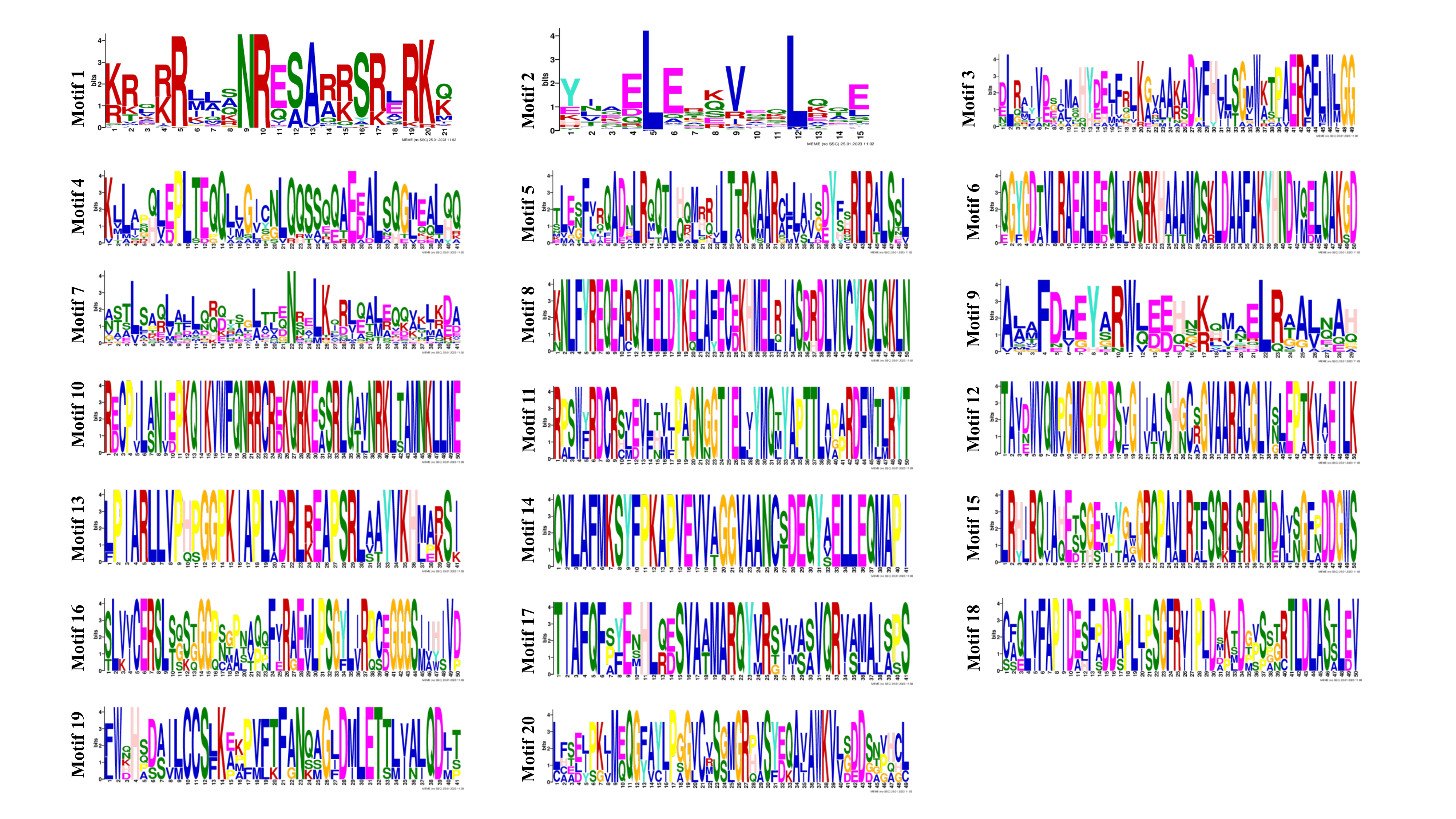

Supplement: Supplementary Figure 2 — Representative logo of the predicted motif (Motif 1- Motif 20) of PgbZIP proteins. [file Image_2.tiff]

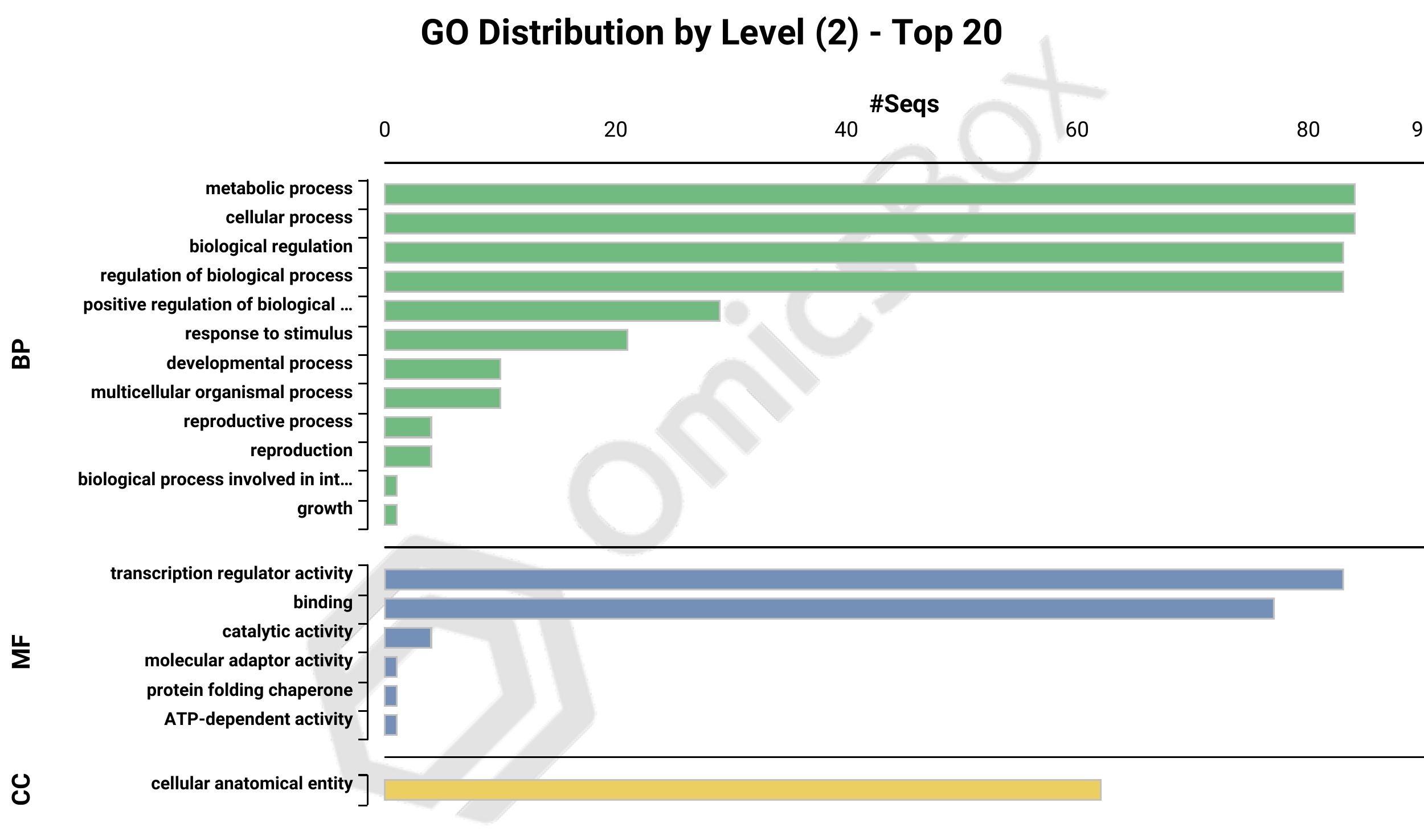

Supplement: Supplementary Figure 3 — The Gene Ontology (GO) analysis defining the biological processes, molecular function and cellular components of PgbZIP proteins by Level (2). [file Image_3.tiff]

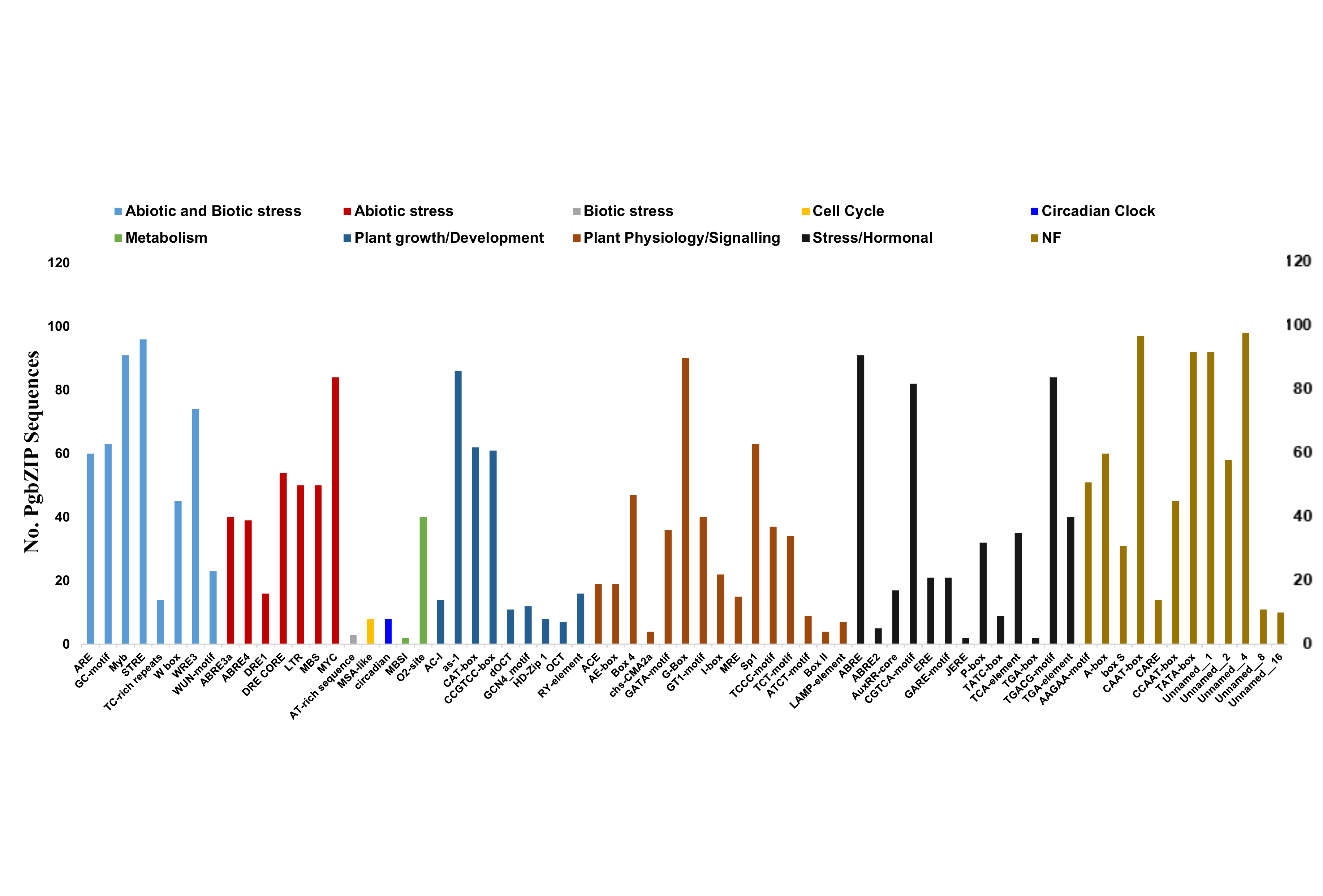

Supplement: Supplementary Figure 4 — Cis-element analysis of PgbZIP genes in 2000 bp upstream region. Color of the bars indicates the group (shown in legend) it belongs. Diverse cis-elements were predicted on the upstream region of PgbZIP genes. [file Image_4.tiff]

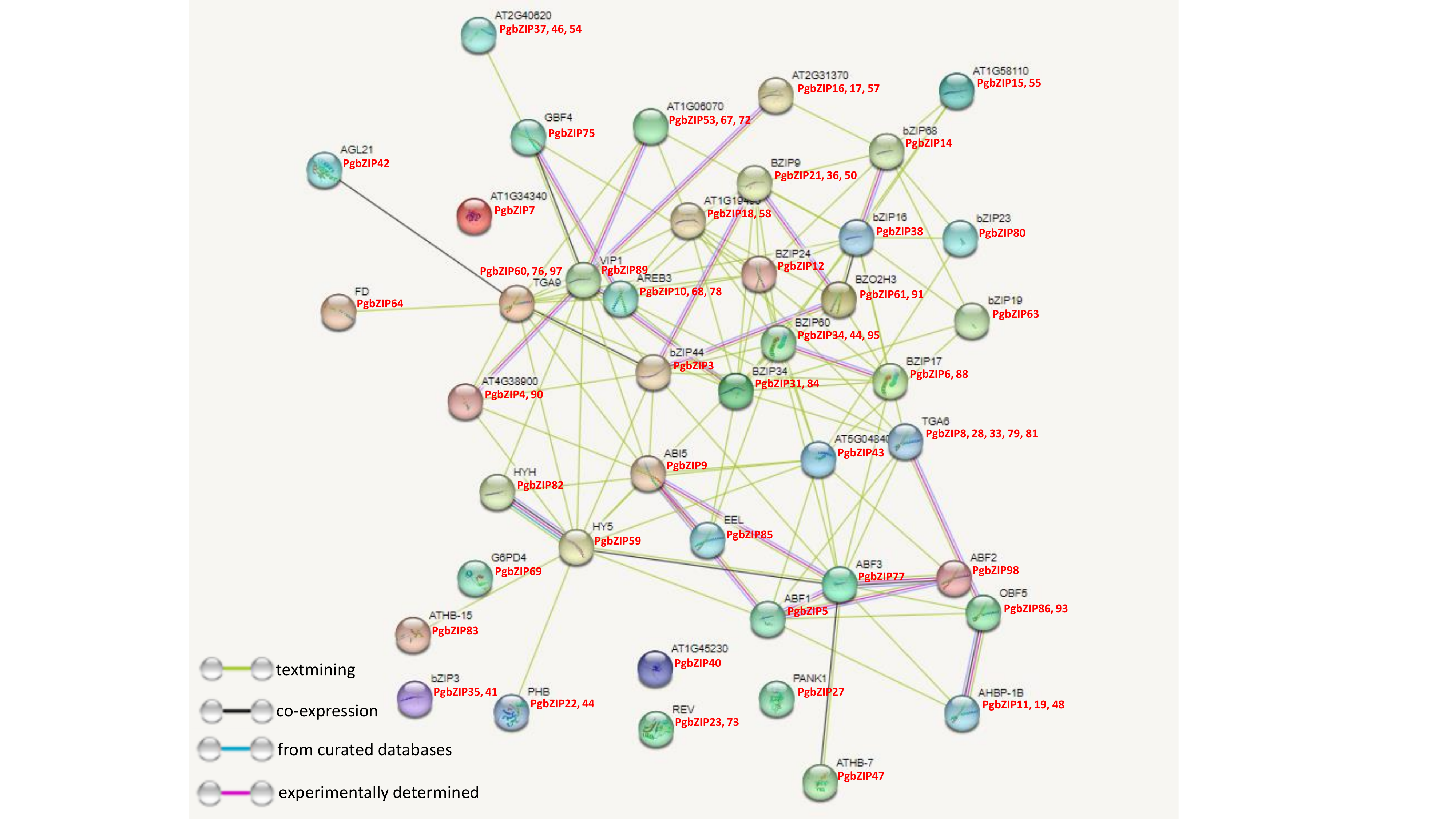

Supplement: Supplementary Figure 5 — Protein-protein interaction network for PgbZIPs based on their orthologs in Arabidopsis. The PgbZIP proteins were shown in the red font below with the Arabidopsis orthologs. The PgbZIP proteins were predicted to have interaction with each other. [file Image_5.tiff]

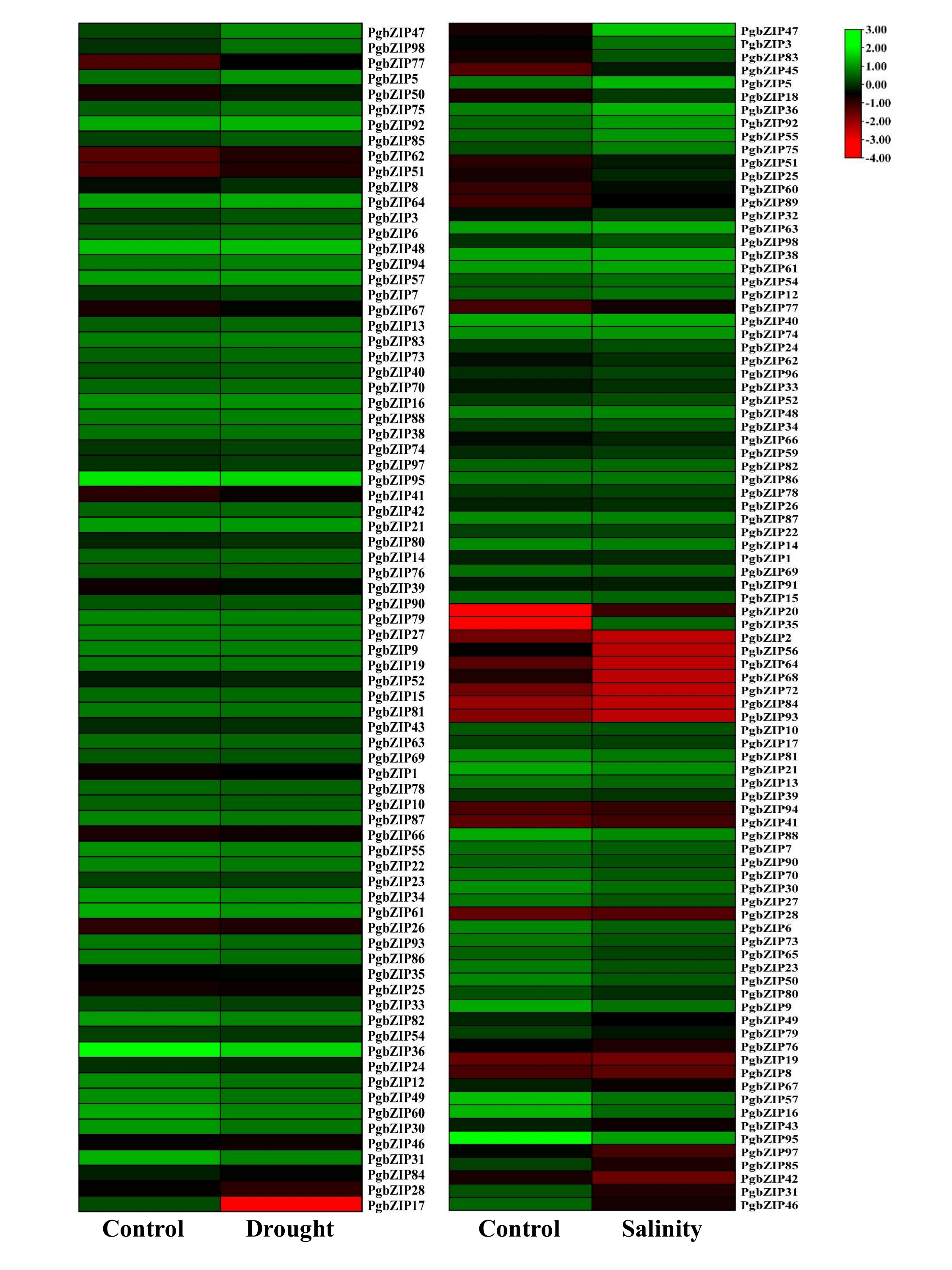

Supplement: Supplementary Figure 6 — In-silico expression analysis of PgbZIP genes under drought and salt stress using publicly available RNA-seq data. The scale bar indicates lower (Red, -4) to higher (Green, 3) expressions level. Differential expression pattern was obtained for PgbZIP genes, [file Image_6.tiff]

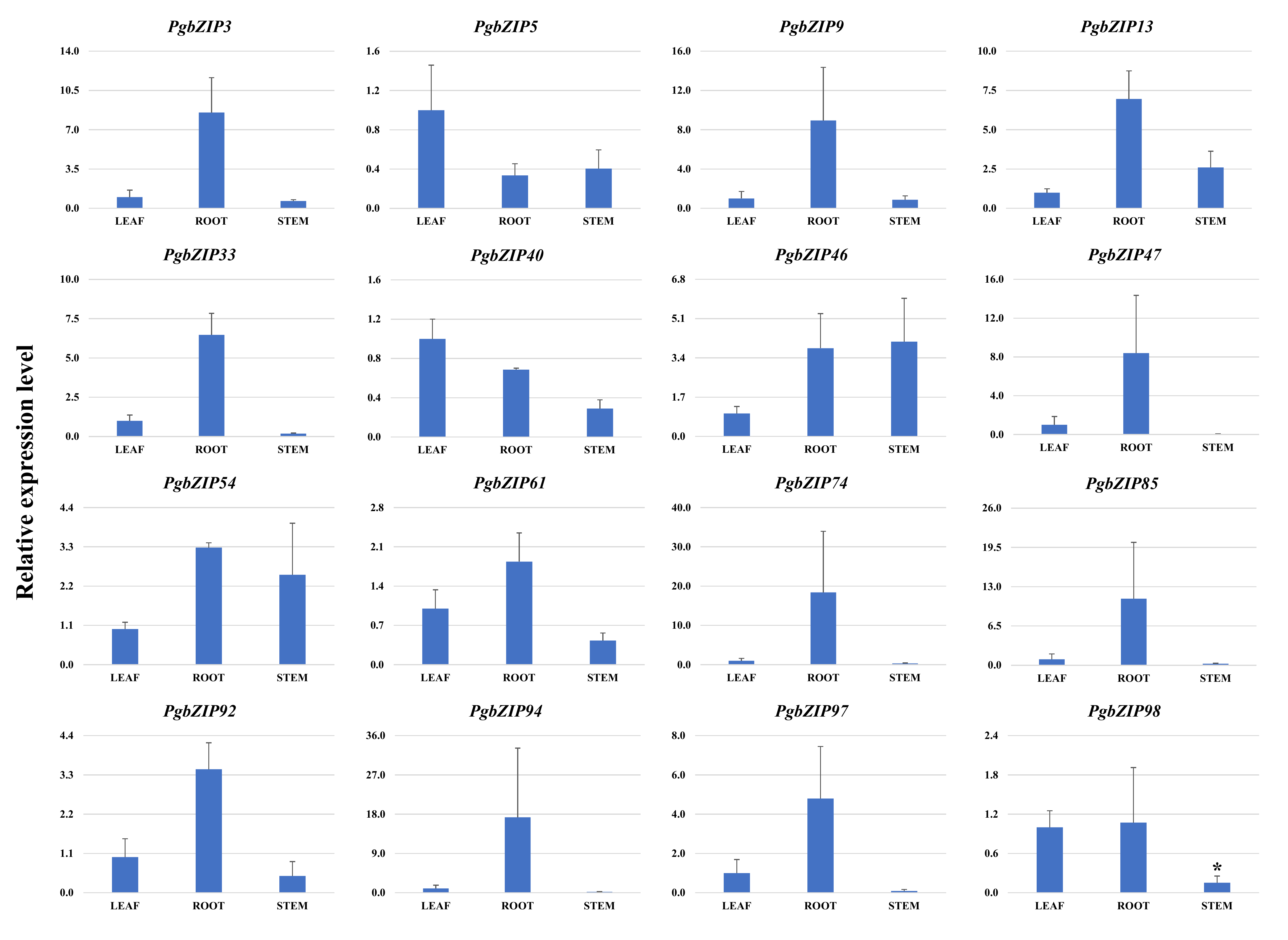

Supplement: Supplementary Figure 7 — Tissue-specific expression analysis of PgbZIP genes in Leaves, Stem and Root tissues of pearl millet. Most of the bZIP genes were highly expressed in the root. The significant difference in the mean is indicated by *P < 0.05, **P < 0.01, as obtained by Student’s t-test. [file Image_7.tif]

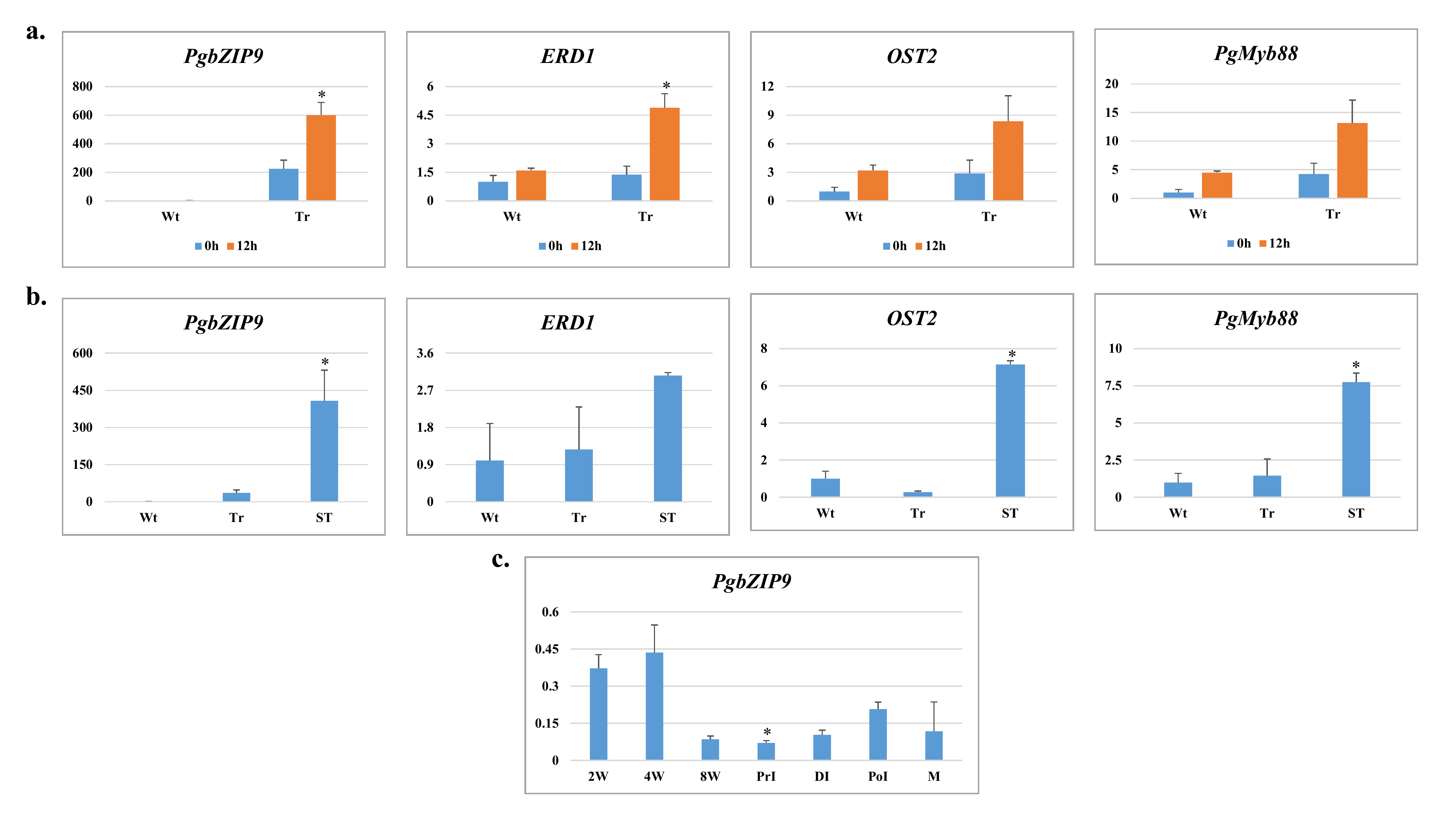

Supplement: Supplementary Figure 8 — (A). Expression level of PgbZIP9 and stress-related genes in transiently overexpressed pearl millet seedlings. Overexpressed seedlings showed higher expression level of PgbZIP9 and stress genes. Wt: Wild type pearl millet seedlings, Tr: Pearl millet seedlings transiently transformed with PgbZIP9 gene (B). The expression level of PgbZIP9 and stress-related genes in transiently overexpressed pearl millet callus. Overexpressed callus showed higher expression level of PgbZIP9 and stress genes. Wt: Wild type pearl millet callus, Tr: Pearl millet callus transformed with PgbZIP9 gene (without stress), ST: Transformed callus treated with 250mM NaCl (C). Expression analysis of PgbZIP9 at different growth of pearl millet including 2-week (2W), 4-week (4W), 8-week (8W), pre-inflorescence (PrI), during-inflorescence (DI), post-inflorescence (PoI) and maturation (M). PgbZIP9 had higher expression level at early stage of life cycle in pearl millet. [file Image_8.tiff]
